# The Shape of a Vehicle Windshield Affects Reaction Time and Brain Activity During a Target Detection Task

**DOI:** 10.3389/fnhum.2020.00183

**Published:** 2020-05-26

**Authors:** Takafumi Sasaoka, Maro G. Machizawa, Yoshihisa Okamoto, Koji Iwase, Toshihiro Yoshida, Nanae Michida, Atsuhide Kishi, Masaki Chiba, Kazuo Nishikawa, Shigeto Yamawaki, Takahide Nouzawa

**Affiliations:** ^1^Brain, Mind, and KANSEI Sciences Research Center, Hiroshima University, Hiroshima, Japan; ^2^Mazda Motor Corporation, Hiroshima, Japan

**Keywords:** attention, optic flow, precuneus, visibility, vehicle design

## Abstract

**Background**: Achieving clear visibility through a windshield is one of the crucial factors in manufacturing a safe and comfortable vehicle. The optic flow (OF) through the windshield has been reported to divert attention and could impair visibility. Although a growing number of behavioral and neuroimaging studies have assessed drivers’ attention in various driving scenarios, there is still little evidence of a relationship between OF, windshield shape, and driver’s attentional efficacy. The purpose of this research was to examine this relationship.

**Methods**: First, we quantified the OF across the windshield in a simulated driving scenario with either of two types of the windshield (a tilted or vertical pillar) at different speeds (60 km/h or 160 km/h) and found more upward OF along the tilted pillar than along the vertical pillar. Therefore, we hypothesized that the predominance of upward OF around the windshield along a tilted pillar could distract a driver and that we could observe the corresponding neural activity. Magnetic resonance scans were then obtained while the subjects performed a visual detection task while watching the driving scene used in the OF analysis. The subjects were required to press a button as rapidly as possible when a target appeared at one of five positions (leftmost, left, center, right, and rightmost).

**Results**: We found that the reaction time (RT) on exposure to a tilted pillar was longer than that on exposure to a vertical pillar in the leftmost and rightmost conditions. Furthermore, there was more brain activity in the precuneus when the pillar was tilted than when it was vertical in the rightmost condition near the pillar. In a separate analysis, activation in the precuneus was found to reflect relative changes in the amount of upward OF when the target was at the rightmost position.

**Conclusions**: Overall, these observations suggest that activation in the precuneus may reflect extraneous cognitive load driven by upward OF along the pillar and could distract visual attention. The findings of this study highlight the value of a cognitive neuroscientific approach to research and development in the motor vehicle manufacturing industry.

## Introduction

When driving a vehicle, we rely mainly on visual information from the outside world (e.g., Booher, 1978; although criticized by Sivak, 1996). For safe driving, accurate recognition of the environment through the windshield is essential. A driver needs to be aware of how a road curves, what its surface is like, and whether or not there is anything to avoid, such as pedestrians, cyclists, or physical obstacles. Therefore, ensuring visibility through the windshield is important when designing a vehicle but in the past has been evaluated subjectively without objective evidence.

Between a driver and the outside world, there is a windshield with “A-pillar,” positioned on either side of the car windshield. This pillar occludes visual information in the outside world, which raises the question of whether or not visibility through the windshield could be improved if the windshield did not have a pillar or if the pillar was transparent. However, in the vehicle development industry, engineers have their empirical opinion that a pillar provides important information that aids steering. For example, steering without a pillar is difficult because the pillar provides spatial cues when steering the vehicle, just like the lines on the road. Moreover, the shape of a windshield is thought to determine the driver’s stereognostic sense of the outside world. When cornering, a windshield that is shaped in a way that occludes the apex of the corner (known as the clipping point) destabilizes the steering maneuver. Therefore, we speculated that the frame of a car windshield would be important for driving and that the shape of the windshield would affect the visibility. However, our evidence for this speculation was solely based on subjective reports from engineers. Therefore, in the motor vehicle manufacturing industry, it would be desirable to design the shape of the windshield that allows clear visibility supported by quantitative assessment.

One way of measuring the clarity of vision while driving would be to test how well drivers can focus their attention without becoming distracted. Driver attention has been of great interest in the applied research fields of ergonomics and psychology. For example, driving performance has been examined in the settings of driver fatigue (Brown, 1994), aging (Wood, 2002), talking (Becic et al., 2010; Atchley and Chan, 2011), and using a cell phone (Briem and Hedman, 1995). Moreover, the role of driver attention in driving (Graydon et al., 2004), the effect of alcohol consumption on brain activity during a simulated driving task (Calhoun et al., 2005), and the influence of conversation on brain activity while driving have been investigated by functional magnetic resonance imaging (fMRI; Just et al., 2008; Uchiyama et al., 2012; Schweizer et al., 2013) and magnetoencephalography (Bowyer et al., 2009). Hsieh et al. (2009) and Palmiero et al. (2019) have published a comprehensive review of the findings of this research. However, a relatively new area of research, known as neuroergonomics, is elaborating on these neuroscientific findings for application in the field of ergonomics (Parasuraman, 2003; Lees et al., 2010; Navarro et al., 2018). Such studies have highlighted the significant role of neuroscientific analysis in real-life situations associated with driving.

**Figure 1:**
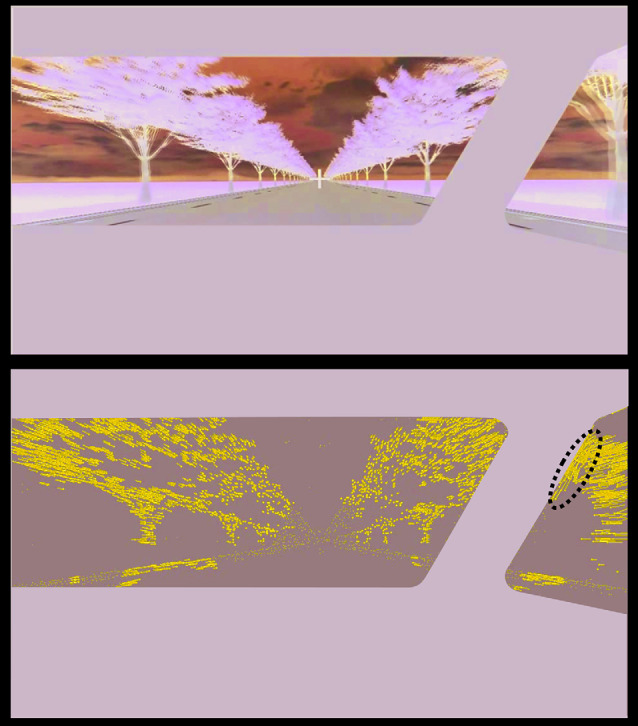
Example of an optic flow (OF) field calculated by the TV-L1 OF estimation method (Sánchez Párez et al., 2013) for a frame of a movie clip onto which a tilted pillar was superimposed. The top panel shows one frame of the motion scenery generated by a driving simulator; a tilted pillar was superimposed onto the movie clip. The bottom panel depicts the OF field calculated for the frame shown in the top panel. The length and orientation of each blue line represent the magnitude and orientation of each of the OF, respectively. Along the right side of the pillar, there are some OFs with an angle that is close to that of the pillar indicated by the dotted line.

The optic flow (OF) along the windshield might contribute to impaired visibility while driving. OF is the distribution of apparent velocities of movement of brightness patterns in an image that is caused by the relative motion of objects and the observer (Horn and Schunck, 1981). Although there has been a good deal of research on attentional effects of various factors while driving, there is limited information on the influence of OF on visual attention in drivers (e.g., Higuchi et al., 2019). Furthermore, how OF affects brain activity is little known. For instance, it has been reported that the distinct brain regions in the V5/MT complex activated to different components of OF (circular and radial motions, vs. translational motion; Morrone et al., 2000). However, the neural substrates for the effect on visuospatial attention of the OF have not been examined. Movement of a vehicle in the drivers’ field of view causes OF that allows the driver to recognize the direction of travel and velocity of the vehicle. It is possible to occlude the field of OF across the windshield with a pillar, which results in ambiguity when estimating motion. This ambiguity is similar to the estimation problems that arise with the “aperture problem” (Nakayama and Silverman, 1988) whereby if an oblique grating is drifting horizontally behind an aperture, we perceive the motion of the grating as ambiguous because the direction of motion cannot be correctly interpreted as a result of occlusion of the ends of the grating. In the case of driver vision, radial OFs emanating from the vanishing point is viewed through a windshield with a pillar. Locally at the intersection point of OF with the pillar, the relationship between the OF and the pillar could be considered as that between a grating in translational motion and the aperture, as in the case of the aperture problem. In a natural viewing situation, where no obstacles such as windshields or pillars interfere with the view, OF emerges as the viewer moves forward or backward. However, when the subject is a driver, the OF is disrupted at the point of occlusion of the windshield by the pillar. In this situation, the angle of the pillar would be an important factor in terms of causing the perception of ambiguous motion. Given that more of the OF intersects obliquely with a tilted pillar than with a vertical pillar, perception of motion close to the tilted pillar would be more ambiguous, resulting in incorrect interpretation of the direction of movement. Such erroneous information may impede the viewer’s interpretation of visual information. In a preliminary experiment, we detected upward OF along the tilted pillar where the OF was obstructed, which was *not* part of the OF in a natural viewing situation (see Figure 1). This finding suggested that occlusion of the field of OF by the pillar induced upward OF, i.e., the tilted pillar caused a situation like an aperture problem. Therefore, it is plausible that a tilted pillar may elicit more upward OF more frequently than a vertical pillar. Moreover, given that OF itself may attract visual attention (Higuchi et al., 2019), this extraneous-OF^1^ may distract the driver from necessary driving operations.

We hypothesized that a tilted pillar might produce extraneous-OF leading to the distraction of attention and that the shape of the windshield might influence activations in the brain regions related to visuospatial attention, putatively associated with OF. To test these hypotheses, we analyzed the OF elicited by two types of the windshield using a tilted pillar and a vertical pillar to determine which type of pillar elicited more upward OF (Experiment 1). After confirming the amount of OF elicited by each type of windshield, we performed an fMRI study to examine the neural responses associated with the effect of the OF around the pillar in which we monitored brain activity in human subjects during a visual attention task (Experiment 2).

## Experiment 1

**Figure 2:**
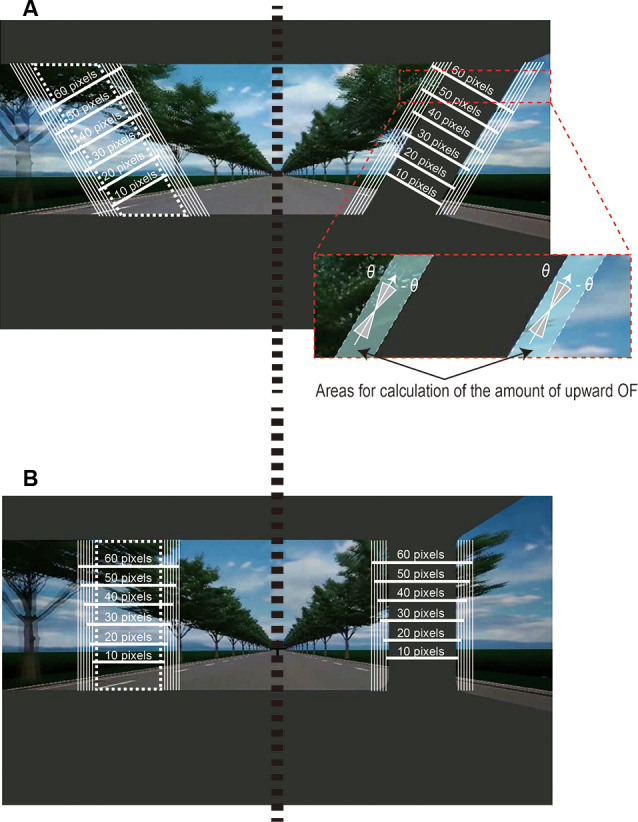
The area used for the analysis of the OF field. We calculated the mean amount of upward OF along the **(A)** tilted or **(B)** vertical pillar on the right side and a “virtual” pillar (white dotted line) on the left side of the windshield positioned symmetrically to the pillar with respect to the vertical line (black dotted line) at the center of the windshield. We defined six cases of areas (*A*) along the pillar from 10 to 60 pixels with a 10-pixel increment. We also defined 16 cases with an angle in the range of −θ to θ (θ = 0°, 1°, …, 15°) to the pillar. In each case of θ and *A*, we summed the magnitude of OFs that had an orientation within an angle range from −θ to θ in the ranges of the area within *A* pixels along the pillar for each frame of the movie clip (the inset figure). The inset figure shows a case in which θ equals 15° and *A* equals 60, i.e., the amount of upward OF was calculated by summing the OF that emerged in the area within 60 pixels along the pillar and had an angle in the range within ±15°. See “Analysis of Optic Flow” section for details.

In this experiment, we examined whether or not there was a difference in the amount of upward OF between two types of the windshield, i.e., one with a tilted pillar and the other with a vertical pillar. If any difference was observed, we further examined at what angle of OF the difference became evident and at what distance from the pillar the difference became apparent. We also measured the amount of upward OF at different speeds because the speed would be a potential cause for the OF along the pillar. We created a movie clip showing the view from the driver’s seat in a vehicle traveling in a straight line at a constant speed using a driving simulator. Based on the Japanese standard of the driver’s seat being on the right side, a tilted or vertical pillar was superimposed on the right side of the windshield with no pillar on the left side. We compared the OF in the field around the pillar on the right side and that in the area symmetric to the invisible vertical line at the center of the windshield on the left side (see Figure 2). We then calculated the difference in upward OF between the areas on the right and left sides of the windshield as an index of the extraneous-OF caused by the pillar. If our hypothesis was correct, the upward OF not originated from the movie clip itself would be more evident along the side of a pillar and more prominent for a windshield with a tilted pillar than one with a vertical pillar.

### Materials

We generated a movie clip using a driving simulator (D3sim, Mitsubishi Precision Co., Ltd., Japan; see Figure 1). The movie clip consists of a driving scene viewed through the windshield of a vehicle traveling straight on a road with surrounding objects, including trees. The same scene was played at a slow speed (60 km/h) and a fast speed (160 km/h). For a windshield, we created a superimposed pattern that consisted of the shades of the dashboard and a pillar to simulate the driver’s view, as shown by the gray area in Figure 1. We determined the angle, width, and size of the pillar based on the actual vehicle of the Mazda Motor Corporation. The angle between the vertical line and the tilted pillar was set at 30° for the tilted pillar whereas the vertical pillar was placed exactly along the vertical line. The movie clip had a resolution of 1920 × 1080 pixels and was 7 min 10 s in duration with a frame rate of 30/s.

### Analysis of Optic Flow

The OF was estimated in each frame of the movie clip by the TV-L1 method (Sánchez Párez et al., 2013) implemented with the OpenCV library (version 2.4.9). We calculated the OF using the “superres::createOptFlow_DualTVL1” function with its default parameters. First, we calculated the orientation and magnitude of the OF for each frame of the movie clip in a windshield with a tilted pillar and a windshield with a vertical pillar. We then summed the magnitude of the OF values that had an orientation within an angle range from −θ to θ (θ = 0°, 1°, …, 15° to the pillar for each frame; see Figure 2). We defined the summed magnitude of OF as the amount of upward OF. We also examined the range of area along the pillar in which upward OF is elicited for each type of pillar (tilted or vertical) by summing the amount of OF with an angle range of −θ to θ within various ranges (*A* = 10, 20, 30, 40, 50, 60 pixels) along the pillar.

To determine the amount of extraneous-OF in the area along the pillar, we calculated the mean amount of OF within the left visual field of the windshield that was positioned symmetrically to the actual pillar on the right side relative to the vertical line at the center of the windshield (Figure 2). We then subtracted the mean amount of OF along the “virtual” pillar from that along the actual pillar for each of four movie clips (slow-vertical, slow-tilted, fast-vertical, and fast-tilted).

Since the OF values were sampled from the movie clip consisting of a driving scene on the road appearing repeatedly surrounding objects, these values could show an auto-correlation. To compare the amounts of OF for the tilted pillar and the vertical pillar at slow and fast speeds, we subjected three θ values (5°, 10°, and 15°) to a four-way non-parametric analysis of variance (ANOVA) using permutation (Anderson, 2001) with the sum of OF as the dependent variable and θ (5°, 10°, 15°), *A* (10, 20, 30, 40, 50, 60 pixels), speed (fast, slow), and pillar (tilted, vertical) as independent factors. To reduce high dimensionality, we made 218-dimensional vectors for each OF time-series of four conditions by averaging OF values for every 60 frames (60 frames corresponds to a repetition time (2 s) of fMRI measurement in Experiment 2). We used the adonis function in the vegan package version 2.4.2^2^ running on R version 3.6.0. Once the overall effects of factors were confirmed, we conducted *post hoc* comparisons using randomization tests with 10,000 repetitions. In the case when both effects of speed and pillar were significant, we conducted multiple pairwise comparisons using randomization tests.

### Results

**Figure 3:**
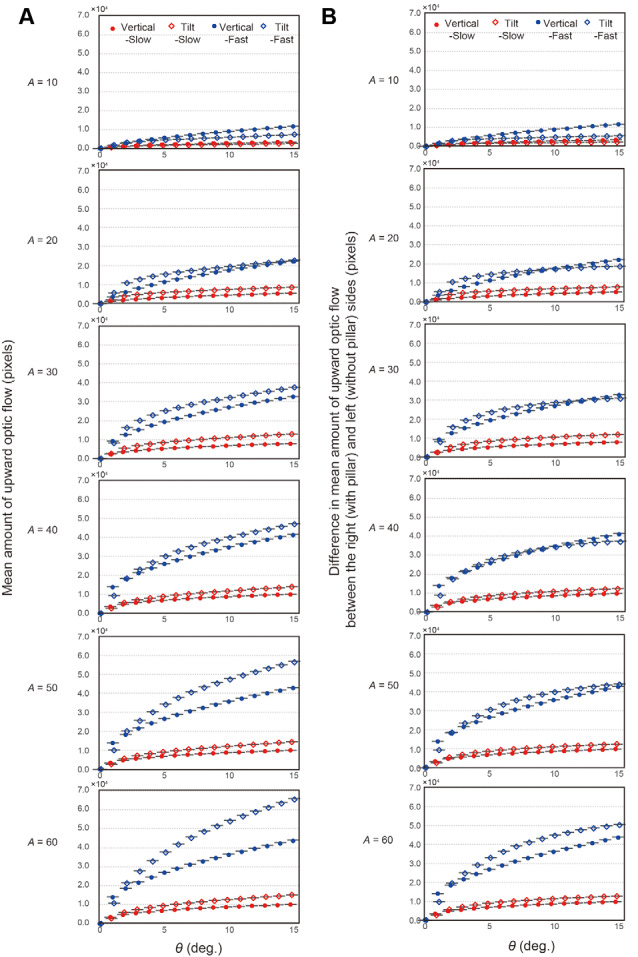
Results of analysis of the field of OF. **(A)** The mean amount of upward OF along the tilted or vertical pillar at a slow or fast speed as a function of the range of the angle of the OF to the pillar (θ) used for calculation of the amount of upward OF. Each row corresponds to each of the six conditions of area (*A*) along the pillar, where the OF was analyzed. Error bars represent standard errors. **(B)** The difference in the mean OF between the left (without a pillar) and right (with a pillar) sides of the windshield as a function of the range of the angle of the OF to the pillar (θ) used for calculation of the amount of upward OF. Each row corresponds to each of six cases on the area along the pillar (*A*), where the OF was analyzed. Error bars represent standard errors.

Figure 3A shows the amount of upward OF for the tilted pillar and the vertical pillar at slow and fast speeds as a function of the range of angle (θ) to the pillar. The qualitative profiles of the upward OF in area (*A*) beyond 20 pixels were similar at all points of interest, indicating that the faster the vehicle speed, the more the upward OF. Four-way non-parametric ANOVA revealed that all factors had significant main effects in a two-way, three-way, and four-way interactions (all *F*-values >29, *p* < 0.001; Supplementary Table S1). After obtaining this result, we performed randomization tests in all cases of θ and *A* to examine the effect of speed and the effect of pillar for comparisons of the sum of OF between slow and fast speed conditions and comparisons between tilted and vertical pillar conditions, respectively. Regarding the effect of speed, the differences between slow and fast speed conditions were significant in all cases of θ and *A* (all *p* < 0.0001 with Bonferroni correction). Regarding the effect of the pillar as well, the differences between tilted and vertical pillar conditions were significant in all cases of θ and *A* (all *p* < 0.0001 with Bonferroni correction). Only on five cases of *A* above 20 pixels, the upward OF was greater for the tilted pillar than for the vertical pillar (Table 1). Multiple comparisons of pillar and speed showed that upward OF was most pronounced for the tilt-fast condition, followed by the vertical-fast, tilt-slow, and vertical-slow conditions (all *p* < 0.0001, randomization tests with Bonferroni correction) except that the difference between the tilt-fast and vertical-fast conditions when A equals 20 was not significant.

**Table 1:** Results of *post hoc* multiple comparisons for the effects of speed and pillar on upward optic flow.

θ	5°		10°		15°	
*A* (pixels)	*p*-value	Cliff’s delta	*p*-value	Cliff’s delta	*p*-value	Cliff’s delta
**Effect of speed (Slow-Fast)**						
10	**0.000**	**−0.295**	**0.000**	**−0.250**	**0.000**	**−0.250**
20	**0.000**	**−0.728**	**0.000**	**−0.637**	**0.000**	**−0.555**
30	**0.000**	**−0.726**	**0.000**	**−0.700**	**0.000**	**−0.681**
40	**0.000**	**−0.676**	**0.000**	**−0.677**	**0.000**	**−0.674**
50	**0.000**	**−0.724**	**0.000**	**−0.731**	**0.000**	**−0.732**
60	**0.000**	**−0.737**	**0.000**	**−0.741**	**0.000**	**−0.742**
**Effect of the pillar (Vertical-Tilted)**						
10	**0.000**	**0.369**	**0.000**	**0.496**	**0.000**	**0.500**
20	**0.000**	**−0.482**	**0.000**	**−0.391**	**0.0006**	**−0.309**
30	**0.000**	**−0.480**	**0.000**	**−0.453**	**0.000**	**−0.435**
40	**0.000**	**−0.389**	**0.000**	**−0.423**	**0.000**	**−0.427**
50	**0.000**	**−0.449**	**0.000**	**−0.480**	**0.000**	**−0.486**
60	**0.000**	**−0.470**	**0.000**	**−0.491**	**0.000**	**−0.495**

*The table shows the p-values, and effect sizes (Cliff’s delta; Cliff, 1993) for speed (fast, slow) and pillar (tilted, vertical) in each of three cases in which the range of the angle of the optic flow to the pillar (*θ*) was set to 5°, 10°, and 15° (column) and in each of six cases for the area (*A*) along the pillar (row). A negative value of Cliff’s delta represents the case in which the effect for the fast speed was larger than for the slow speed, or in which the effect for the windshield with a tilted pillar was larger (shown shaded in the table). When the effect was statistically significant [*p*-value was smaller than the Bonferroni-corrected *p*-threshold (0.05/18 = 0.002778)], the values are shown in bold*.

Figure 3B shows the mean difference in OF between the left (without a pillar) and right (with a pillar) sides of the windshield in each of six cases of *A*, representing extraneous-OF caused by the pillar. Four-way non-parametric ANOVA with the difference in OF as the dependent variable and θ, *A*, speed, and pillar as independent variables again revealed that the main effects of all factors and two-way, three-way, and four-way interactions were significant (all *F*-values > 11, *p* < 0.001; Supplementary Table S2). Regarding the *post hoc* comparisons between slow and fast speed conditions, randomization tests revealed that the differences between slow and fast speed conditions were significant both in the tilted and vertical pillar conditions in all cases of θ and *A* (all *p* < 0.0001 with Bonferroni correction), indicating that the amount of extraneous-OF was greater at the fast speed than at the slow speed in all cases of θ and *A*. Regarding the *post hoc* comparisons between tilted and vertical pillar conditions, the amount of extraneous-OF was greater for the windshield with a tilted pillar than for the windshield with a vertical pillar when *A* equals 30, 50, and 60 and when θ equals 5 and 10, and when *A* equals 20 and when θ equals 5°. When θ was 15°, the windshield with a tilted pillar elicited more extraneous-OF when *A* was 60 (Table 2). For the cases in which the effect of the pillar was significant, we checked the significance for multiple comparisons using the randomization test. At the slow speed, we observed that more upward OF was elicited by the windshield with a tilted pillar than by the windshield with the vertical pillar when *A* was above 20 (all *p* < 0.0001, randomization test with Bonferroni correction) in all cases of θ. At the fast speed, the extraneous-OF was elicited more by the windshield with a tilted pillar when *A* equals 20, 30, 50, and 60 when θ was 5° (all *p* < 0.0001 with Bonferroni correction). When θ was 10°, the tilted pillar elicited more extraneous-OF than the vertical pillar when *A* was 30, 50, and 60 (all *p* < 0.0001 with Bonferroni correction). When θ was 15°, the tilted pillar caused more extraneous-OF than the vertical pillar when *A* was 60 (*p* < 0.0001 with Bonferroni correction). When *A* was 10, the vertical pillar elicited more extraneous-OF than the tilted pillar in all cases of θ, regardless of speed (*p* < 0.0001 with Bonferroni correction).

**Table 2:** Results of *post hoc* multiple comparisons for the effects of speed and pillar on the difference in upward optic flow between the left (without pillar) and right (with pillar) sides of the windshield.

θ	5°		10°		15°	
*A* (pixels)	*p*-value	Cliff’s delta	*p*-value	Cliff’s delta	*p*-value	Cliff’s delta
**Effect of speed (Slow—Fast)**						
10	**0.000**	**−0.259**	**0.000**	**−0.249**	**0.000**	**−0.249**
20	**0.000**	**−0.712**	**0.000**	**−0.508**	**0.000**	**−0.307**
30	**0.000**	**−0.706**	**0.000**	**−0.593**	**0.000**	**−0.422**
40	**0.000**	**−0.586**	**0.000**	**−0.498**	**0.000**	**−0.368**
50	**0.000**	**−0.665**	**0.000**	**−0.639**	**0.000**	**−0.545**
60	**0.000**	**−0.701**	**0.000**	**−0.700**	**0.000**	**−0.661**
**Effect of the pillar (Vertical—Tilted)**						
10	**0.000**	**0.452**	**0.000**	**0.498**	**0.000**	**0.500**
20	**0.000**	**−0.465**	0.003	−0.261	0.402	−0.061
30	**0.000**	**−0.459**	**0.000**	**−0.347**	0.176	−0.179
40	0.015	−0.272	0.241	−0.209	0.460	−0.080
50	**0.0001**	**−0.356**	**0.001**	**−0.351**	0.087	−0.256
60	**0.000**	**−0.404**	**0.000**	**−0.421**	**0.0004**	**−0.380**

*The table shows the p-values, and effect sizes (Cliff’s delta) for speed (fast, slow) and pillar (tilted, vertical) in each of three cases in which the range of the angle of the optic flow to the pillar (*θ*) was set to 5°, 10°, and 15° (column) and in each of six cases for the area (*A*) along the pillar (row). A negative value of Cliff’s delta represents the case in which the effect for the fast speed was larger than for the slow speed, or in which the effect for the windshield with a tilted pillar was larger (shown shaded in the table). When the effect was statistically significant [*p*-value was smaller than the Bonferroni-corrected *p*-threshold (0.05/18 = 0.002778)], the values are shown in bold*.

## Experiment 2

Participants in this experiment performed a task in which they needed to detect a visual target presented to them while watching the simulated driving scene used in Experiment 1 and having their brain activity measured on fMRI. It was predicted that: (1) the reaction time (RT) when detecting the target would be longer when the target was presented around a tilted pillar than around a vertical pillar; and (2) depending on the impact of the task, extraneous activation in the region of the brain involved in attention control would be observed during detection of the target while the subject performed the detection task through the windshield with a *tilted* pillar.

### Materials and Methods

#### Participants

Thirty-five healthy subjects (16 male, 19 female, aged 19–52 years, mean ± SD = 28.9 ± 8.6) participated in the experiment. All study participants were right-handed except for one male subject. The fMRI data, as well as behavioral data, for three female subjects, were excluded from the analysis because of excessive body movement (>4 mm) during scanning. For subsequent analysis, one subject being left-handed was also rejected for behavioral data analysis that concerns the handedness, see details below. The study was approved by the Research Ethics Committee of Hiroshima University (approval number E-965-3). All study participants provided written informed consent before enrolment in the study.

#### Stimulus

**Figure 4:**
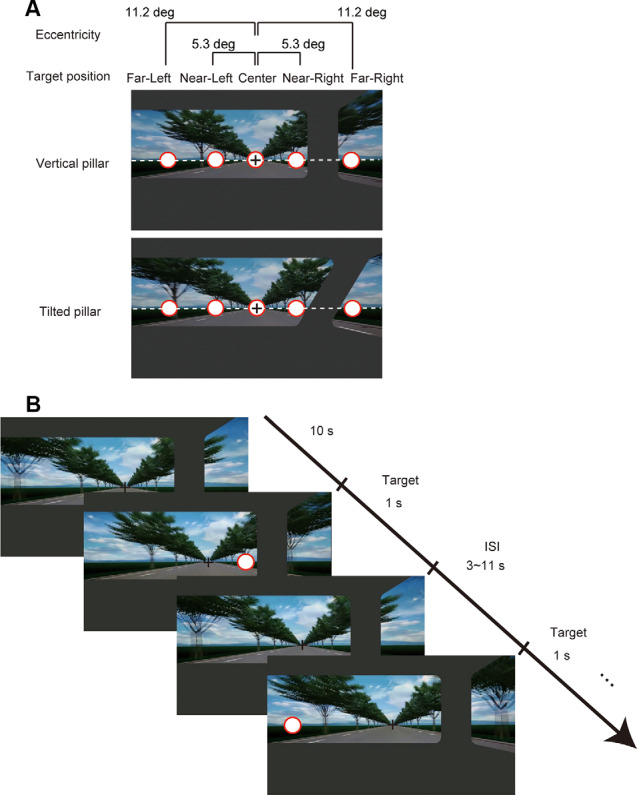
**(A)** Target positions on the display for the two-pillar conditions. The task was to detect a target that appeared on the display at one of the five positions (i.e., far-left, near-left, center, near-right, and far-right from the leftmost position). **(B)** Example task time course of the first two trials. The task started with 10 s of continuous motion pictures of scenery with a fixation cross at the center of the display. A target appeared for 1 s at one of the five positions at random with a varied inter-stimulus interval in the range of 3–11 s. Either a tilted or a vertical pillar was superimposed on the movie clip throughout one experimental block. See “Materials and Methods” section for details.

We used the same movie clip as that shown in the OF analysis in Experiment 1 as a background image sequence. The movie clip was presented on an MRI-compatible 32-inch LCD monitor (NordicNeuroLab, Bergen, Norway) with a resolution of 1,920 × 1,080 pixels that subtended 17.4° × 30.4° visual angles. The target to be detected was a white disk with a red border that was presented for 1 s at randomized intervals and in the far-left, near-left, center, near-right, and far-right from the leftmost position (Figure 4). The inter-stimulus interval was randomized between 3 and 11 s. The timing of the target presentation in this stochastic design was optimized using the Optseq2 software (Dale, 1999). The size of the target was 1.3° visual angles. The target positions for far-left/right and near-left/right were fixed at 11.2° and 5.3° visual angles, respectively, to the left or right away from a central fixation point (0°) along the horizontal line. The distance from the tilted pillar to the near-right target was 0.33° visual angles (21 pixels), and that to the far-right target was 0.11° visual angles (seven pixels). The distance from the vertical pillar to the near-right target was 0.32° visual angles (20 pixels), that to the far-right target was 0.47° visual angles (30 pixels).

#### Task Procedures

The subjects watched for the appearance of the stimulus through an angled mirror placed on a head coil. When a target appeared, they were required to respond as rapidly as possible by pressing a button using their right thumb irrespective of the position of the target and to maintain fixation on the fixation point presented throughout the task. Before performing the main task, all subjects performed a practice session with a total of 10 targets for 30 s. There were four sessions per subject. During each session, the targets were presented 100 times (20 times for each target position). The target position was selected randomly for each presentation. As a behavioral measure of attention, we analyzed the RTs for each subject and the accuracy of target detection (hit or miss) at each position.

#### Experimental Design

We used a factorial block design with two conditional factors, i.e., vehicle speed (60 or 160 km/h) and pillar (vertical and tilt) and a target position factor (far-left, near-left, center, near-right, and far-right) for each condition. The subjects attended four sessions (7 min 26 s per session), each of which presented one of four (slow-vertical, fast-vertical, slow-tilted, or fast-tilted) conditions. The order of the four conditions was counterbalanced across the study participants.

#### Behavioral Data Analysis

Given the task being a simple target detection exercise, each subject’s responses to a given target were classified as hit or miss, with a missed condition recorded as no record of an RT response; therefore, the mean RT was the average value of correct-only responses. For RT and the number of correct responses, we conducted three-way repeated-measures ANOVA with factors of vehicle speed, pillar, and target position. The modified Shaffer method was used for *post hoc* analysis. Since motor RTs may be faster in the dominant hand compared to non-dominant, one left-handed subject was excluded from the RT analysis. Moreover, we performed the same analysis after excluding three female subjects excluded from the fMRI analysis and one left-handed subject to examine whether the same tendency was kept or not.

#### MRI Acquisition

The fMRI data were acquired using a 3.0-T MRI scanner (Magnetom Verio, Siemens Healthineers, Erlangen, Germany) with an echo-planar T2*-weighted gradient-echo sequence and the following scan parameters: repetition time, 2,000 ms; echo time, 24 ms; 30 slices; 4-mm thickness without gap; voxel size, 3 × 3 × 4 mm; and field of view, 192 mm. A total of 223 volumes were acquired in each condition for each subject. We also acquired an anatomical scan for co-registration purposes using three-dimensional T1-weighted magnetization-prepared rapid gradient-echo imaging (repetition time 2,300 ms; echo time, 2.98 ms; 176 slices; thickness, 1 mm; voxel size, 1 × 1 × 1 mm; the field of view, 256 mm; matrix, 256 × 256).

#### MRI Data Processing and Analysis

Image processing and the statistical analyses were performed using SPM8 software (Wellcome Department of Cognitive Neurology, London, UK^3^). The first five volumes were discarded to allow for T1 equilibration effects. The remaining 218 volumes were spatially realigned to the first of the volumes, re-realigned to the mean of all images to correct for head movement, and a slice-timing correction was performed. T1-weighted anatomical images were co-registered to the first of the echo-planar images (EPIs). The co-registered anatomical images were spatially normalized to the Montreal Neurological Institute template. Parameters derived from this normalization process were then applied to each functional image. The normalized EPIs were spatially smoothed by an 8-mm full width at half maximum Gaussian kernel. The voxel-based statistical analysis on the pre-processed EPIs was performed using the general linear model. The blood oxygenation level-dependent (BOLD) response related to target detection was modeled as a box-car function for the onset of presentation of a target convolved with a canonical hemodynamic response function and then used as a covariate. Six head motion parameters derived from the realignment process were also modeled out to reduce the motion-related artifacts. Before the regression analysis, the low-frequency confounding effects were removed using a high-pass filter with a 128-s cut-off period; serial correlations between scans were estimated using a first-order autoregressive model to remove the variance that could be explained by the previous scans. Regression coefficients for each event were computed for each subject using a fixed-effects model and then taken into group analysis using a random-effects model with a one-sample *t*-test. In the group analysis, we also added the subjects’ age and sex as nuisance parameters to the model. We report the activated brain regions that survived our statistical threshold set at uncorrected *p* < 0.001 at peak level and family-wise error (FWE) corrected *p* < 0.05 at the cluster level.

#### Analysis of OF-Related Brain Activity

**Figure 5:**
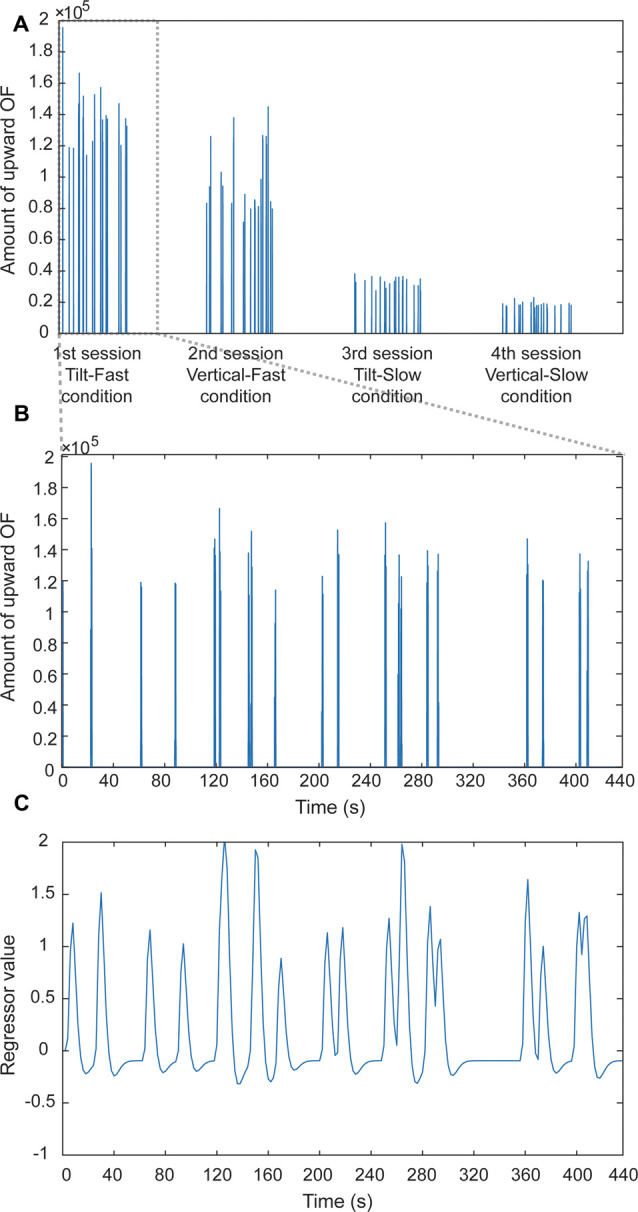
An example of the computational procedure used for an OF-related regressor. **(A)** Amount of upward OF plotted as a function of time (all four sessions in an entire experiment). Each vertical line represents an amount per scanned volume. **(B)** Enlarged view of the amount of upward OF for the first session. **(C)** A regressor was calculated by convolving the time series of the amount of upward OF with a canonical hemodynamic function and down-sampled at 0.5 Hz (equal to repetition time). We carried out two types of normalization of the amount of upward OF, i.e., normalization in each session and across all sessions. See “MRI Data Processing and Analysis” section for details.

Having predicted that upward OF would affect performance, we examined the brain regions activated in relation to upward OF when the target was presented. To address this issue, we performed a regression analysis with a target onset-specific OF regressor. Based on the results of the OF analysis in Experiment 1, we used the time series of the amount of upward OF within an angle of 5° to the pillar summed in the area within 20 pixels along the pillar, given that the effect size of the main effects of the pillar and the speed was the largest on comparison of the extraneous-OF (see Table 2). For each target position, the time series of the amount of upward OF was obtained using the amount of upward OF itself, calculated for each frame in which the target appeared. Also, we examined the OF-related brain activity by performing a regression analysis with an OF regressor. To assess whether the target onset-specific OF-related activity (“Target with OF”) was simply related only with target-detection (“Target-only”) or it still retains the impact of extraneous-OF, we compared the upward OF-related brain activity at all target positions (“Target with OF”) with the upward OF-related brain activity (“OF-only”). This “Target-only” contrast (“Target with OF” > “OF only”) was used as an *exclusive* mask for additional analysis to examine neural activation specific to the extraneous-OF derived at the position of interest. We performed two types of normalization of the upward OF time series to derive different perspectives. For the first analysis, the OFs were Z-score-normalized in each session for each subject to elucidate the brain activity associated with the relative changes in upward OF for each condition. For the other analysis, OFs were Z-score-normalized across all four sessions to examine the brain activity associated with the change in upward OF, this time considering the differences in amount across conditions. Next, the normalized time series were convolved with the canonical hemodynamic function for each normalized OF time series and down-sampled to a repetition time-frequency of 0.5 Hz (corresponding to a repetition time of 2,000 ms) for subsequent regression analysis. The resulting covariate of upward OF was submitted for regression analysis of BOLD signals under each condition (Figure 5). For each speed and pillar condition, regression coefficients for OF regressor and target onset-specific OF regressor for each target position were computed for each subject using a fixed-effects model and then taken into group analysis using a random-effects model with a one-sample *t*-test. In the group analysis, we also added the subjects’ age and sex as nuisance parameters to the model.

### Results

#### Behavioral Data

**Figure 6:**
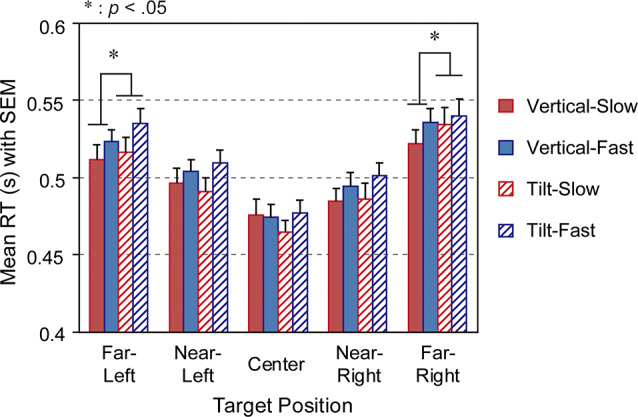
Mean reaction times (RTs) at each target position for types of speed and pillar. The error bars represent the SEM. SEM, standard error of the mean.

For RT, three-way repeated-measures ANOVA with factors of vehicle speed, pillar, and target position for each condition revealed significant main effects of target position (*F*_(2.73,90.04)_ = 97.24; partial η^2^ = 0.747; *p*_corrected_ < 0.001; Greenhouse-Geisser correction was applied to control for violation of the sphericity assumption; Supplementary Table S3) and speed (*F*_(1,33)_ = 10.93, partial η^2^ = 0.249, *p* < 0.005). There was no significant main effect of pillar (*F*_(1,33)_ = 1.11, partial η^2^ = 0.033, *p* = 0.299). *Post hoc* analyses revealed significant differences in RT for all pairs of target positions (all *t*-values_(132)_ > 2.52, all *p-*values < 0.05; Figure 6). *Post hoc* analysis for the main effect of speed revealed that the mean RT for the fast speed was longer than that for the slow speed (*t*_(33)_ = −3.31, *p* < 0.005). In addition to the main effects, a two-way interaction between the factors of pillar and target position was significant (*F*_(3.09,101.89)_ = 3.25, partial η^2^ = 0.090, *p*_corrected_ < 0.05). However, two-way interactions between target position and speed, and between speed and pillar, and a three-way interaction were not significant (target position and speed: *F*_(3.12,102.80)_ = 1.774, *p*_corrected_ = 0.155; speed and pillar: *F*_(1,33)_ = 0.696, *p* = 0.410; three-way interaction: *F*_(3.28,108.23)_ = 1.737, *p*_corrected_ = 0.159). Analyses of simple effects revealed that the difference in the mean RTs between the tilted and vertical pillar conditions was significant at the rightmost (far-right) target position just outside of the pillar and the leftmost (far-left) target position that was the counterpart for the far-right position (far-right: *F*_(1,165)_ = 4.204, partial η^2^ = 0.113, *p* < 0.05; far-left: *F*_(1,165)_ = 3.937, partial η^2^ = 0.107, *p* < 0.05; Figure 6) but at no other positions. *Post hoc* analyses revealed that the mean RT for the tilted pillar condition was longer than that for the vertical condition both at the far-right and far-left target positions (far-right: *t*_(165)_ = 2.050, *p* < 0.05; far-left: *t*_(165)_ = 1.984, *p* < 0.05; two-tails tests).

An additional ANOVA was carried out (*N* = 31; Supplementary Table S4) after excluding one left-handed subject and three subjects with large body movement kept the same tendency as the analysis with *N* = 34. The two-way interaction between the factors of pillar and target position was marginally significant (*F*_(2.99,89.57)_ = 2.59, partial η^2^ = 0.079, *p*_corrected_ = 0.058), and the difference in the mean RTs between the tilted and vertical pillar conditions was marginally significant at the far-right and far-left positions (far-right: *t*_(150)_ = 1.750, *p* = 0.082; far-left: *t*_(150)_ = 1.781, *p* = 0.077; two-tails tests).

Another three-way repeated-measures ANOVA performed for the numbers of correct responses revealed a significant main effect of target position (*F*_(3.39,115.21)_ = 4.381, partial η^2^ = 0.114, *p*_corrected_ < 0.01) but no interactions. *Post hoc* analyses revealed that, out of 20 trials per position, the mean (and standard deviation) number of correct responses was greater at the center (19.77 ± 0.59) than at the most peripheral positions, i.e., far-left and far-right (19.58 ± 0.91 vs. 19.47 ± 0.86; center—far-left, *t*_(34)_ = 3.834, *p* < 0.01; center—far-right, *t*_(34)_ = 3.479, *p* < 0.01). There was no significant difference in the number of correct responses between the far-left and far-right positions (*t*_(34)_ = 1.314, *p* = 0.198). We conducted an additional ANOVA (*N* = 31) after excluding one left-handed subject and three subjects with large body movement for the mean number of correct responses. This kept the same tendency as that of *N* = 35; ANOVA revealed a significant main effect of target position (*F*_(3.00,90.11)_ = 3.692, partial η^2^ = 0.110, *p*_corrected_ < 0.05) and *post hoc* analyses revealed that the mean number of correct responses was greater at the center than at the most peripheral positions (center—far-left, *t*_(30)_ = 3.248, *p* < 0.05; center— far-right, *t*_(30)_ = 3.166, *p* < 0.05; two-tails tests).

#### MRI Data

##### Brain Activity Depending on the Angle of the Pillar and Target Positions

**Figure 7:**
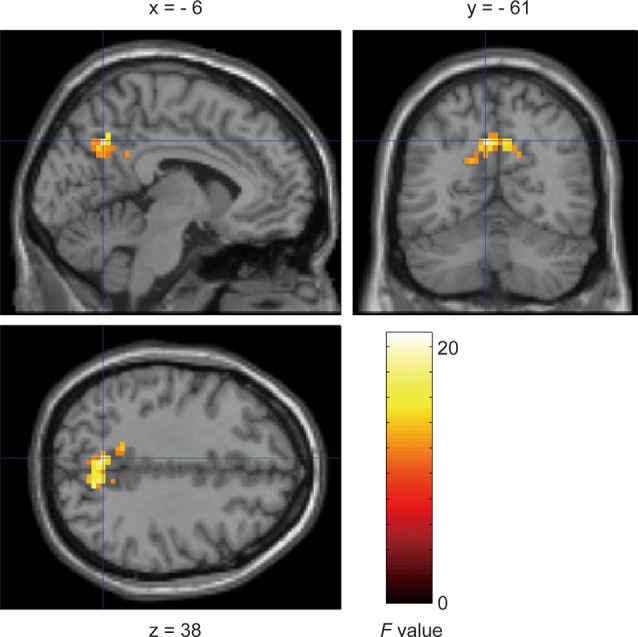
Significant activation observed in the precuneus (BA7/31) bilaterally with an interaction between type of pillar and target position (family-wise error corrected *p* < 0.05 at cluster level). Uncorrected *p*-level at *p* < 0.001 (peak voxels) was applied only for display purposes.

To examine brain activity according to the angle of the pillar, we performed a three-way repeated-measures ANOVA on the BOLD response to the presentation of the target with factors of vehicle speed, pillar, and target position (far-left and far-right, at which a significant or marginally significant simple main effect of the pillar was observed in the behavioral RT analyses, as reported above). This analysis revealed a significant interaction between the pillar and target position in the precuneus bilaterally (BA7/31; Figure 7; Table 3). Moreover, we compared the brain activity for the tilted and vertical pillars when a target was presented at the far-right position. This again revealed that the cluster including the precuneus was significantly activated more for the tilted pillar than for the vertical pillar (Supplementary Figure S1 and Supplementary Table S5). The main effect of the target position was observed in the visual areas bilaterally, i.e., the area extending from the left middle temporal gyrus to the superior parietal lobule and the left precentral gyrus (Table 4). We observed no significant main effects or interactions in other regions (all *F*-values < 11.09).

**Table 3:** Results of functional magnetic resonance imaging of anatomical regions, peak voxel coordinates, and *F*-values for activations showing a significant interaction between pillar and target position.

Anatomical region	Brodmann area	Cluster size	MNI coordinates (mm)			*F*-value
			x	y	z	
White matter		139	−18	−55	22	20.96
Left precuneus	31		−6	−61	38	19.62
Right precuneus	7		12	−67	38	17.68

*Uncorrected *p* < 0.001 at peak level, family-wise error corrected *p* < 0.05 at cluster level. MNI, Montreal Neurological Institute*.

**Table 4:** Results of functional magnetic resonance imaging of anatomical regions, peak voxel coordinates, and *F*-values for activations showing a significant main effect of target position.

Anatomical region	Brodmann area	Cluster size	MNI coordinates (mm)			*F*-value
			*x*	*y*	*z*	
Left middle temporal cortex	39	1446	−42	−76	10	99.73
Left middle occipital gyrus	19		−45	−73	2	82.32
Left middle occipital gyrus	19		−30	−79	22	77.36
Right lingual gyrus	19	495	27	−64	−10	86.07
Right middle temporal gyrus	19		45	−64	2	76.18
The right parieto-occipital transition area	19		27	−76	22	20.79
Left precentral gyrus	6	248	−39	−4	50	35.81
Left middle frontal gyrus	6		−24	5	54	24.47
Left precentral gyrus	9		−39	8	30	24.08
Right cuneus	17	95	9	−73	6	25.09
Right inferior parietal lobule	40	53	30	−46	54	21.66
Right superior parietal lobule	7		27	−52	46	20.33
Right superior parietal lobule	7		24	−58	54	18.27

*Uncorrected *p* < 0.001 at peak level, family-wise error corrected *p* < 0.05 at cluster level. MNI, Montreal Neurological Institute*.

We further compared the brain activities between the far-left and far-right positions only for the tilted pillar condition in which excessive OF was dominantly observed. This analysis revealed that the left-lateralized clusters in the occipito-parietal areas, including the precuneus, and the left premotor cortex were more active when the target was presented at the far-right position than at the far-left position. In contrast, the right-lateralized cluster in the fusiform and middle temporal gyri was more active when the target was presented at the far-left position than at the far-right position (Supplementary Figure S2 and Supplementary Table S6).

##### Upward Optic Flow-Related Activity

Given the behavioral results when the RT for target detection was influenced by the angle of the pillar at the far-right position near the pillar where the upward OF was elicited, we focused on the brain activity correlated with OF during the presentation of the target at this position.

**Figure 8:**
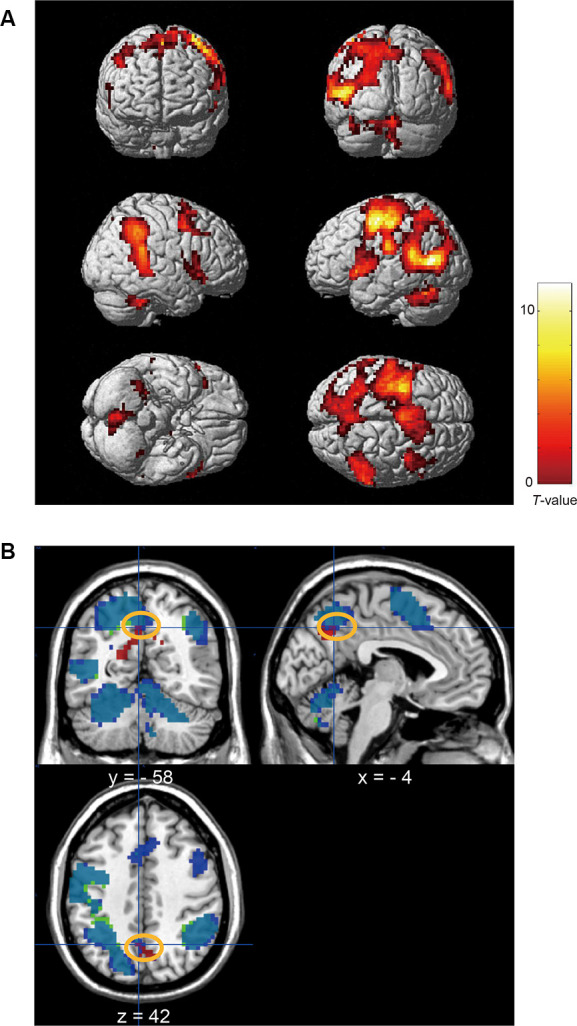
**(A)** Brain activation associated with the relative amount of upward OF (normalized in each condition) that is common in all speed (fast/slow) and pillar (tilted/vertical) conditions when the target was presented at the far-right position (uncorrected *p* < 0.001 at peak level, FWE corrected *p* < 0.05 at cluster level). **(B)** Overlapping activation within the precuneus (the area indicated by an orange line) between the tilted pillar-related and upward OF-related activity when the target was presented at the far-right position (uncorrected *p* < 0.001 at peak level, FWE corrected *p* < 0.05 at cluster level). Red: activated regions showing the interaction between pillar and target position. Blue: regions commonly activated in all four conditions (including speed and types of the pillar) in the analysis of brain activity related to the relative amount of upward OF normalized in each condition when the target was presented at the far-right position (Table 5). Green: regions commonly activated in all four conditions in analysis of brain activity related to the amount of upward OF normalized across all four conditions when the target was presented at the far-right position (Supplementary Table S1).

First, we examined the brain activity associated with the relative change in upward OF for each condition using the upward OF with the regressor Z-score normalized in each session. A two-way repeated-measures ANOVA in which the factors were speed and pillar revealed significant activations that were common to all conditions in the left middle temporal cortex, left precentral gyrus, left lingual gyrus, cerebellum, right superior temporal gyrus, right inferior parietal lobule, left central and frontal operculum, and left opercular portion of the inferior frontal gyrus (Figure 8A; Table 5). Notably, the activated cluster in the parietal cortex overlapped with the area in the precuneus found to be active when the target was presented at the far-right position in the tilted pillar condition (Figure 8B). However, no significant activation indicating any main effect or interaction was observed.

**Table 5:** Results of functional magnetic resonance imaging of anatomical regions, peak voxel coordinates, *t*-values for activations related to a relative change in the amount of upward optic flow (normalized in each condition) that is common in all speed and pillar conditions.

Anatomical region	Brodmann area	Cluster size	MNI coordinates (mm)			*F*-value
			*x*	*y*	*z*	
Left middle temporal cortex	19	3,771	−48	−64	10	11.55
Left precentral gyrus	6		−39	−4	54	9.67
Left superior parietal lobule	7		−27	−52	50	7.62
Left lingual gyrus	19	1,380	−24	−61	−14	8.89
Left cerebellum			−27	−52	−22	7.84
Right cerebellum			18	−52	−22	7.29
Right superior temporal gyrus	22	651	63	−40	18	6.90
Right supramarginal gyrus	40		42	−52	42	5.56
Right supramarginal gyrus	40		45	−49	34	5.51
Right middle frontal gyrus	6	129	48	5	46	4.70
Right middle frontal gyrus	8		45	14	42	3.81
Right middle frontal gyrus	8		42	29	38	3.65
Right inferior frontal gyrus	44	97	57	17	14	4.02
Right inferior frontal gyrus	13		48	2	14	3.87
Right precentral gyrus	44		48	5	6	3.72

*The positive effect of conditions: uncorrected *p* < 0.001 at peak level, family-wise error corrected *p* < 0.05 at cluster level when the target was presented at the far-right position. MNI, Montreal Neurological Institute*.

**Figure 9:**
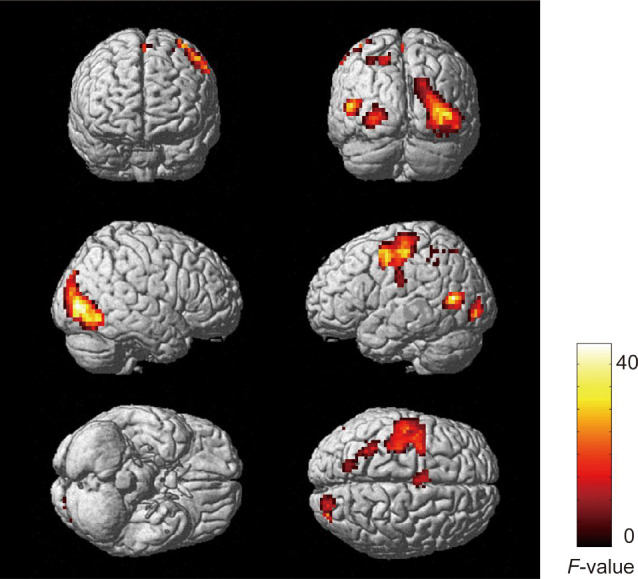
Brain activation associated with the amount of upward OF normalized across all four conditions showing the main effect of fast/slow speed (uncorrected *p* < 0.001 at peak level, FWE corrected *p* < 0.05 at cluster level).

Next, we examined the brain activity related to changes in the amount of upward OF with the regressor Z-score normalized across four sessions. A two-way repeated-measures ANOVA revealed significant main effects of speed in the visual cortex, fusiform gyri, left middle temporal cortex, left pre/postcentral gyri, supplementary motor area, left superior parietal lobule, right inferior parietal lobule, and cerebellum (Figure 9; Table 6). However, there was no significant main effect of speed in the precuneus region, indicating that the precuneus would be active irrespective of speed when the upward OF was elicited. However, activations that were common across all conditions in the left middle temporal cortex, left precentral gyrus, left lingual gyrus, cerebellum, right superior temporal gyrus, right inferior parietal lobule, left central and frontal operculum, and left opercular part of the inferior frontal gyrus overlapped with those obtained using the upward OF regressor Z-score normalized in each session (Supplementary Table S7). The cluster in the parietal cortex, including the precuneus, was close to the activated region when the target was presented at the far-right position in the *tilted* pillar condition (see Figure 8B for overlap). However, we observed no upward OF-related brain responses indicating a significant main effect of pillar or interaction.

**Table 6:** Results of functional magnetic resonance imaging of anatomical regions, peak voxel coordinates, and *F*-values for activations related to the amount of upward optic flow normalized across all four conditions when the target was presented at the far-right position showing the main effect of speed.

Anatomical region	Brodmann area	Cluster size	MNI coordinates (mm)			*F*-value
			*x*	*y*	*z*	
Right inferior occipital gyrus	18	458	36	−82	−2	44.2
Right inferior occipital gyrus	19		39	−70	−10	37.42
Right inferior temporal gyrus	37		48	−64	−10	34.06
Left middle temporal gyrus	19	99	−48	−67	10	39.14
Left inferior occipital gyrus	18	67	−27	−88	−6	30.68
Left middle frontal gyrus	6	372	−33	−1	50	27.44
Left postcentral gyrus	1		−48	−16	54	25.37
Left postcentral gyrus	1		−54	−22	54	25.09
Left superior parietal lobule	7	142	−30	−52	46	26.29
Left superior parietal lobule	7		−15	−70	54	19.47
Left inferior parietal lobule	40		−36	−40	42	17.37
Left supplementary motor area	6	54	−6	2	62	20.09

*Uncorrected *p* < 0.001 at peak level, family-wise error corrected *p* < 0.05 at cluster level. MNI, Montreal Neurological Institute*.

Furthermore, to assure the observed BOLD response (as in Figure 8A) is not simply reflecting the detection of a target but also induced by the extraneous-OF, we compared the upward OF-related activity with target onsets at the far-right position (Figure 8A) and “Target-only” activation. The “Target-only” activation regardless of position was obtained by contrasting the OF-related activity with target onsets (“Target with OF”) with the brain activity for upward OF (“OF-only”). The OF-related activity with target onsets at the far-right position was masked out by the “Target-only” (“Target with OF” > “OF-only”) contrast and it revealed activations in the left parieto-occipital cluster *including* the precuneus and MT/V5, as well as the left superior/middle frontal cortex, and the supplementary motor area. Finally, when we compare the resultant contrast map with the activation for the target onset at the far-right position specifically in the *tilted* pillar condition, there was a notable overlap in the precuneus within the left parieto-occipital cluster (Supplementary Table S8 and Supplementary Figure S3). Therefore, the precuneus activity was not simply explained by the target detection, instead, it was confirmed that the additional activation in the precuneus region is likely associated with the extraneous-OF.

## Discussion

In this study, we hypothesized that the tilted pillar would cause extraneous-OF that lead to the distraction of the driver’s attention. The OF analysis in Experiment 1 confirmed that upward OFs with relatively small angles to the pillar (at around 5°) were elicited more in the windshield with a tilted pillar than in the one with a vertical pillar, particularly at around the edges of the pillar. To examine the potential impact of the extraneous-OF on visual attention, each subject’s brain activity was monitored while performing the visual target-detection task in Experiment 2. As predicted, the RTs for detection of the target were influenced by the type of the windshield, namely the angle of the pillar. The RT was longer with the tilted pillar than with the vertical pillar when detecting a target presented at the far-right and far-left positions. Our fMRI analyses suggested that the activity in the precuneus was possibly related to the excessive OF along a pillar that may lead to additional attentional load.

### Extraneous-Optic Flow Elicited in the Tilted Pillar

At the slow speed, the upward extraneous-OF was observed more along the tilted pillar than along the vertical pillar in most of the cases of areas and angles. At the fast speed, the tilted pillar elicited more upward extraneous-OF with relatively small angles to the pillar than the vertical pillar. This suggests that an OF with a large angle (e.g., more than 10°) to the *tilted* pillar approached the OF values in the movie clip without a pillar because an angle with which OFs radiating from the vanishing point intersects the pillar becomes smaller for the tilted pillar than for the vertical pillar. At the fast speed, the total OFs increase, thereby the upward extraneous-OF with a relatively large angle to the tilted pillar decreased by the subtraction of the upward OF on the side without a pillar. These results suggest that upward extraneous-OF with relatively small angles to the pillar was the most pronounced. The mechanism accounting for this finding might be the same as that with the barber pole illusion (Wallach, 1935) whereby an oblique grating moving horizontally behind an elongated rectangular aperture leads to an illusory perception of upward motion along the major axis of the aperture. Given that actual OFs in a driving scene intersect with smaller angles to the tilted pillar than to the vertical pillar, this could result in more OFs intersecting obliquely with the tilted pillar than with the vertical pillar, leading to a situation similar to that of the barber pole illusion. Therefore, it was speculated that illusory perception of upward motion, which has a small angle to the pillar, could occur around the tilted pillar.

While more upward OF is elicited with a vertical pillar than a tilted pillar when the angle is extremely small (a θ of 1°), the subtle effect could be negligible for the interpretation of behavioral and neuroimaging data. This upward OF is almost in parallel to the pillar, a large object (e.g., a tree) standing parallel to the vertical pillar might result in the computation of OF when it passes behind the pillar. However, the trend of more OF for the vertical pillar flips above an angle of 1° when considering sufficient area to calculate the amount of OF (more than 10 pixels). Nevertheless, it is known that the spatial resolution of our peripheral vision is lower than that of the foveal vision. In our case, the receptive field sizes of far-left/right target positions (at 12° visual angles away from the fixation cross) are approximately 2–4° in V1, 8–10° in V4 (as reported in Gattass et al., 1981). Since the area of consideration here at 10-pixels (equivalent to 0.16° visual angles) would be little to consider in peripheral vision, the different tendencies within the area might not influence the behavioral and neuroimaging results for our case of peripheral attention.

To note, the effect of speed was more dominant for the amount of upward OF than the angle of the pillar. Nevertheless, the type of pillar did affect the amount of upward OF. Because the primary purpose of this study was to examine the influence of the windshield and its pillar for the application of vehicle designing, we focused primarily on the difference between the tilted and vertical pillars. Taking all results on OF analyses together, we adopted the case of *A* equals 20, and θ equals 5° in that both the effects of pillar and speed were largest for the subsequent analysis of OF-related brain activity.

### The Effect of the Angle of the Pillar on the Reaction Time for Target Detection

The results of OF analysis suggested that there were considerable differences in OF between the two types of the windshield and that it would be plausible that the extraneous-OF especially at around the pillar could distract attention during driving. Given the assumption that OF may diminish attentional performance around the pillar, we conducted a visual target-detection task.

Focused analysis of the RT for detection of the target at the far-left and far-right positions, which were equidistant from the fixation point, indicated that the RT was longer when a target appeared near to the pillar (far-right) than when it was presented far from the pillar (far-left). However, human spatial attention is reportedly better in the left visual field than in the right visual field due to the hemispherically specific role of visuospatial attention in the right hemisphere (Pouget and Driver, 2000; Rees et al., 2000; for review see Shulman et al., 2010). This could prompt speculation about our findings because the pillar was only placed within the right visual hemifield; however, a direct comparison between the right and left visual field conditions suggests that this might not be the case. In the comparison of mean RTs focused on the far-most target positions (far-left, far-right), the tilted pillar tends to slow the RT more than the vertical pillar irrespective of visual hemifield (see Figure 6). These results suggest that excessive induction of OF along the pillar might impede the instantaneous detection of a target. Therefore, these behavioral data support our hypothesis that the extraneous-OF along the tilted pillar distracts the driver’s visuospatial attention.

In the current study, however, the angles of the pillar and the distance between the near/far-right target positions and the pillar was not fully equated. The distance at the far-right target position for the tilted pillar was within 10 pixels (seven pixels corresponding to 0.11° visual angles), but the far-right position for the vertical pillar was 30 pixels (0.47° visual angles) apart from the pillar. Again, there may be of a critical point at purely perceptual study at fovea; however, the difference of the two pillars of less than 1° visual angle in the distance might not have influenced target detection because of the low spatial resolution in the peripheral vision (e.g., Gattass et al., 1981). A careful investigation may be necessary to reveal as to how the distance between the target and the pillar impact on RT and brain activity for target detection in a future study.

Moreover, the main effect of speed on the RT was more dominant than the angle of the pillar. Since we did not observe any interaction of speed with target position and pillar, the effect of speed was common in all target positions and all pillar types. Therefore, our result suggests that the effect of speed is not related to the existence of the pillar that we focused on in the present study.

### Activation in the Precuneus Related to Target Detection for the Windshield With a Tilted Pillar

In the fMRI analysis, we observed that the precuneus was activated when the target was presented at the far-right position on the windshield with a tilted pillar, in which the RT for target detection was increased. Given the finding that more upward OFs were elicited in the windshield with a tilted pillar than in the one with a vertical pillar, increased activation in the precuneus may be involved in the slowed RTs putatively caused by upward extraneous-OF. The precuneus has been reported to be fundamental to attentional functions. Simon et al. (2002) showed that the precuneus is more activated during a visual attention task than during calculation and other visuospatial tasks related to grasping, pointing, and saccade. Several studies have suggested that the precuneus is involved in attentional shift (Le et al., 1998; Astafiev et al., 2003). It has also been reported that the precuneus is activated in tasks involving covert shifts of spatial attention (Gitelman et al., 1999; Beauchamp et al., 2001). In contrast, abnormal activation in the precuneus is thought to be related to attention deficit hyperactivity disorder, implying a deficit in sustained attention (Castellanos et al., 2008; Christakou et al., 2013). In the present study, we observed increased activation in the precuneus related to the detection of a target around the tilted pillar. In the windshield with a tilted pillar, the extraneous-OF was elicited irrespective of the speed revealed by OF analysis in Experiment 1. These results may reflect a redundant attentional shift for target detection presented around the tilted pillar.

However, there could be another interpretation of the precuneus activity that reflected the saccadic effects since the subjects might suppress the urge to look towards the target. This view is supported by the previous research showing that the frontoparietal areas including the precuneus are involved in saccadic suppression (Brown et al., 2007). However, this possibility might be ruled out by the fact that the precuneus was active more for the far-right target detection than for the far-left target detection in the tilted pillar condition while the saccadic effect would be same for the far-left and far-right targets that are in the equidistance to the fixation cross. Thus, this result would provide the support that the precuneus reflected the extraneous-OF elicited around the tilted pillar near the far-right target that accompanies a subtle impact on visual attention.

### Overlapping Activation in the Precuneus Related to the Relative Change in Upward Optic Flow and Target Detection in the Windshield With a Tilted Pillar

To examine whether the relative change in upward OF for each speed and pillar condition affected brain activity, we first examined the upward OF-related brain activation using the upward OF regressor Z-score normalized in each session. This revealed that brain activity was related to upward OF when the participants detected the target presented at the far-right position in the left middle temporal area, premotor areas, and the bilateral and medial parietal areas including the precuneus. The previously reported Talairach coordinates of the human middle temporal area (*x* = −38, *y* = −74, *z* = 8; Zeki et al., 1991), which is a brain region important for motion perception, were included in the activated cluster. This suggests the perception of OFs created by micro-flows of visual fractions recruits brain regions responsible for the detection of motion, while a salient distinct object is not necessarily moving. To detect the target presented at the far-right position, the participants had to shift their attention to the right peripheral visual field. Therefore, it is reasonable that activation in the middle temporal area might be lateralized to the left. Furthermore, activation in the left motor cortices could be caused by the motor response to the target using their right hand.

More interestingly, the activated cluster related to the upward OF in the precuneus partly overlapped with that showed activation related to the target detection presented at the far-right position in the windshield with a tilted pillar (see Figure 8B). The second analysis using the upward OF regressor that was Z-score normalized across four sessions revealed that activity in the precuneus did not show the main effect of speed. This finding suggests that the precuneus was active irrespective of the speed, which co-varied with the absolute amount of upward OF. Therefore, the precuneus activity for the target detection of the far-right position where the upward OF was the most prominent was not influenced by the *absolute* amount of upward OF, but by the *relative* amount of upward OF in a particular speed-and-pillar condition.

Moreover, a comparison of the “Target with OF” at the far-right position and the “Target-only” contrasts still showed activation in the precuneus, supporting the precuneus activity may not simply be related to target detection. Overall, these observations suggest that the upward OF might be related to additional attentional load, which might invoke activations in the precuneus. However, there remains the possibility that the precuneus activation observed in the OF-related activity with target-onsets at the far-right position that was masked out by the “Target-only” contrast still reflected the difference in visuospatial processing across different target positions. It is because that the “Target-only” contrast was indirectly derived from the comparison of the upward OF-related brain activity at all target positions and the upward OF-related brain activity throughout the task (“OF-only”). Because of our experimental constraint that the background movie clip was present throughout the task, the “Target-only” activity could have been only obtained by the subtraction of “OF-only” brain activity from “Target with OF” activity. This is certainly a limitation of the current study. A future study with a target-detection task with *no* background movie clip shall rule out the possibility as mentioned above by achieving the direct comparison between “Target with OF” and “Target-only” at the target position of interest.

### Limitations

Our neurobehavioral results provide quantitative evidence that supports the validity of evaluation based on the attentional loading of the driver when assessing visibility through a windshield. However, the sample size of this study (35 subjects) is relatively small for an fMRI study for generalization.

In the present study, we determined the shape and angles of the pillar based on the actual vehicle in production. However, it is possible that the various parameters, such as the width of the pillar, would affect the results. Furthermore, only one angle of tilt was examined in this study. To apply our results to the design of vehicles, systematic variations of the parameters of the shape of a pillar and windshield may be needed in future studies.

More importantly, the experimental environment in the MRI scanner is markedly different from the actual driving environment because of constraints in MRI measurement, such as posture in the scanner and the view through the small mirror attached to the head coil in front of the participant. Measurements of brain activity when subjects are driving a real vehicle using wearable brain imaging techniques, such as electroencephalography, may be necessary (e.g., Protzak and Gramann, 2018) to elaborate our findings to realistic situations. Moreover, our experiment focused on a simple situation in which a vehicle was traveling straight in one direction. Examinations in other driving situations, e.g., along a winding road or turning at intersections), would be required.

## Conclusion

We have found that activation in the precuneus is associated with an increased RT for the detection of a target on a windshield with a tilted pillar. The precuneus activation for detection of the target presented outside the tilted pillar in the periphery was also influenced by the relative change in extraneous-OF in the visual field. These results provide behavioral and neuroscientific evidence that the task (driving)-irrelevant OFs along the pillar are responsible for the excessive attentional shift. Finally, our study was a neuroscientific investigation that provides rich insights for the design of safe vehicles, at least for the windshield.

## Data Availability Statement

The data are available from the corresponding author upon reasonable request and with the permission of the ethics committee.

## Ethics Statement

The studies involving human participants were reviewed and approved by Research Ethics Committee of Hiroshima University. The patients/participants provided their written informed consent to participate in this study.

## Author Contributions

TS, YO, KI, TY, NM, AK, MC, KN, and TN conceived and designed the experiments. TS and YO performed the experiments and analyzed the data. TY contributed to the OF analysis. TS, MM, and SY contributed to the writing of the manuscript.

## Conflict of Interest

YO, KI, TY, NM, AK, MC, KN, and TN are employed by the company Mazda Motor Corporation. YO, TY, AK, MC, KN, and TN are inventors of the “Vehicle view adjustment device” (JP 6311524) used in this study and are the patent holders. The remaining authors declare that the research was conducted in the absence of any commercial or financial relationships that could be construed as a potential conflict of interest.

## Footnotes

^1^Here, we define the additionally induced OF on the top of naturally occurring OF as “extraneous-OF.”

^2^https://www.rdocumentation.org/packages/vegan/versions/2.4-2

^3^www.fil.ion.ucl.ac.uk/spm

## Abbreviations

ANOVA: analysis of variance
FWE: family-wise error
MRI: magnetic resonance imaging
OF: optic flow
RT: reaction time.

## References

[B1] Anderson M. J. (2001). A new method for non-parametric multivariate analysis of variance. Austral Ecol. 26, 32–46. 10.1111/j.1442-9993.2001.01070.pp.x

[B2] Astafiev S. V. Shulman G. L. Stanley C. M. Snyder A. Z. Van Essen D. C. Corbetta M. (2003). Functional organization of human intraparietal and frontal cortex for attending, looking, and pointing. J. Neurosci. 23, 4689–4699. 10.1523/JNEUROSCI.23-11-04689.2003 12805308 PMC6740811

[B3] Atchley P. Chan M. (2011). Potential benefits and costs of concurrent task engagement to maintain vigilance: a driving simulator investigation. Hum. Factors 53, 3–12. 10.1177/0018720810391215 21469529

[B4] Beauchamp M. S. Petit L. Ellmore T. M. Ingeholm J. Haxby J. V. (2001). A parametric fMRI study of overt and covert shifts of visuospatial attention. NeuroImage 14, 310–321. 10.1006/nimg.2001.0788 11467905

[B5] Becic E. Dell G. S. Bock K. Garnsey S. M. Kubose T. Kramer A. F. (2010). Driving impairs talking. Psychon. Bull. Rev. 17, 15–21. 10.3758/pbr.17.1.15 20081155

[B6] Booher R. H. (1978). Effects of visual and auditory impairment in driving performance. Hum. Factors 20, 307–320. 10.1177/001872087802000306 680693

[B7] Briem V. Hedman L. R. (1995). Behavioural effects of mobile telephone use during simulated driving. Ergonomics 38, 2536–2562. 10.1080/00140139508925285

[B305] Brown I. D. (1994). Driver fatigue. Hum. Factors 36, 298–314. 10.1177/001872089403600210 8070794

[B8] Brown M. R. Vilis T. Everling S. (2007). Frontoparietal activation with preparation for antisaccades. J. Neurophysiol. 98, 1751–1762. 10.1152/jn.00460.2007 17596416

[B304] Bowyer S. M. Hsieh L. Moran J. E. Young R. A. Manoharan A. Liao C. C. J.. (2009). Conversation effects on neural mechanisms underlying reaction time to visual events while viewing a driving scene using MEG. Brain Res. 1251, 151–161. 10.1016/j.brainres.2008.10.001 18992728 PMC2741688

[B9] Calhoun V. D. Carvalho K. Astur R. Pearlson G. D. (2005). Using virtual reality to study alcohol intoxication effects on the neural correlates of simulated driving. Appl. Psychophysiol. Biofeedback 30, 285–306. 10.1007/s10484-005-6384-0 16167192

[B300] Castellanos F. X. Margulies D. S. Kelly C. Uddin L. Q. Ghaffari M. Kirsch A.. (2008). Cingulate-precuneus interactions: a new locus of dysfunction in adult attention-deficit/hyperactivity disorder. Biol. Psychiatry 63, 332–337. 10.1016/j.biopsych.2007.06.025 17888409 PMC2745053

[B10] Christakou A. Murphy C. M. Chantiluke K. Cubillo A. I. Smith A. B. Giampietro V.. (2013). Disorder-specific functional abnormalities during sustained attention in youth with attention deficit hyperactivity disorder (ADHD) and with autism. Mol. Psychiatry 18, 236–244. 10.1038/mp.2011.185 22290121 PMC3554878

[B11] Cliff N. (1993). Dominance statistics: ordinal analyses to answer ordinal questions. Psychol. Bullet. 114, 494–509. 10.1037/0033-2909.114.3.494

[B12] Dale A. M. (1999). Optimal experimental design for event-related fMRI. Hum. Brain Mapp. 8, 109–114. 10.1002/(sici)1097-0193(1999)8:2/3<109::aid-hbm7>3.0.co;2-w 10524601 PMC6873302

[B301] Gattass R. Gross C. G. Sandell J. H. (1981). Visual topography of V2 in the macaque. J. Comp. Neurol. 201, 519–539. 10.1002/cne.902010405 7287933

[B302] Gitelman D. R. Nobre A. C. Parrish T. B. LaBar K. S. Kim Y. H. Meyer J. R.. (1999). A large-scale distributed network for covert spatial attention: further anatomical delineation based on stringent behavioural and cognitive controls. Brain 122, 1093–1106. 10.1093/brain/122.6.1093 10356062

[B13] Graydon F. X. Young R. Benton M. D. Genik R. J. Posse S. Hsieh L. (2004). Visual event detection during simulated driving: identifying the neural correlates with functional neuroimaging. Transp. Res. Part F Traffic Psychol. Behav. 7, 271–286. 10.1016/j.trf.2004.09.006

[B14] Higuchi Y. Inoue S. Hamada H. Kumada T. (2019). Artificial optic flow guides visual attention in a driving scene. Hum. Factors [Epub ahead of print]. 10.1177/0018720819847022 31125278

[B15] Horn B. K. P. Schunck B. G. (1981). Determining optical flow. Artif. Intell. 17, 185–203. 10.1016/0004-3702(81)90024-2

[B16] Hsieh L. Young R. A. Bowyer S. M. Moran J. E. Genik R. J. II. Green C. C.. (2009). Conversation effects on neural mechanisms underlying reaction time to visual events while viewing a driving scene: fMRI analysis and asynchrony model. Brain Res. 1251, 162–175. 10.1016/j.brainres.2008.10.002 18952070

[B17] Just M. A. Keller T. A. Cynkar J. (2008). A decrease in brain activation associated with driving when listening to someone speak. Brain Res. 1205, 70–80. 10.1016/j.brainres.2007.12.075 18353285 PMC2713933

[B303] Le T. H. Pardo J. V. Hu X. (1998). 4 T-fMRI study of nonspatial shifting of selective attention: cerebellar and parietal contributions. J. Neurophysiol. 79, 1535–1548. 10.1152/jn.1998.79.3.1535 9497430

[B18] Lees M. N. Cosman J. D. Lee J. D. Fricke N. Rizzo M. (2010). Translating cognitive neuroscience to the driver’s operational environment: a neuroergonomic approach. Am. J. Psychol. 124, 391–411. 10.5406/amerjpsyc.123.4.0391 21291157 PMC3268652

[B19] Morrone M. C. Tosetti M. Montanaro D. Fiorentini A. Cioni G. Burr D. C. (2000). A cortical area that responds specifically to optic flow, revealed by fMRI. Nat. Neurosci. 3, 1322–1328. 10.1038/81860 11100154

[B20] Nakayama K. Silverman G. H. (1988). The aperture problem-I. Vision Res. 28, 739–746. 10.1016/0042-6989(88)90052-1 3227650

[B21] Navarro J. Reynaud E. Osiurak F. (2018). Neuroergonomics of car driving: a critical meta-analysis of neuroimaging data on the human brain behind the wheel. Neurosci. Biobehav. Rev. 95, 464–479. 10.1016/j.neubiorev.2018.10.016 30442593

[B22] Palmiero M. Piccardi L. Boccia M. Baralla F. Cordellieri P. Sgalla R.. (2019). Neural correlates of simulated driving while performing a secondary task: a review. Front. Psychol. 10:1045. 10.3389/fpsyg.2019.01045 31143148 PMC6521777

[B23] Parasuraman R. (2003). Neuroergonomics: research and practice. Theor. Issues Ergon. Sci. 4, 5–20. 10.1080/14639220210199753

[B24] Pouget A. Driver J. (2000). Relating unilateral neglect to the neural coding of space. Curr. Opin. Neurobiol. 10, 242–249. 10.1016/s0959-4388(00)00077-5 10753799

[B25] Protzak J. Gramann K. (2018). Investigating established EEG parameter during real-world driving. Front. Psychol. 9:2289. 10.3389/fpsyg.2018.02289 30532722 PMC6265363

[B26] Rees G. Wojciulik E. Clarke K. Husain M. Frith C. Driver J. (2000). Unconscious activation of visual cortex in the damaged right hemisphere of a parietal patient with extinction. Brain 123, 1624–1633. 10.1093/brain/123.8.1624 10908192

[B27] Sánchez Párez J. Meinhardt-Llopis E. Facciolo G. (2013). TV-L1 optical flow estimation. Image Process. Line 3, 137–150. 10.5201/ipol.2013.26

[B28] Schweizer T. A. Kan K. Hung Y. Tam F. Naglie G. Graham S. (2013). Brain activity during driving with distraction: an immersive fMRI study. Front. Hum. Neurosci. 7:53. 10.3389/fnhum.2013.00053 23450757 PMC3584251

[B29] Shulman G. L. Pope D. L. W. Astafiev S. V. McAvoy M. P. Snyder A. Z. Corbetta M. (2010). Right hemisphere dominance during spatial selective attention and target detection occurs outside the dorsal frontoparietal network. J. Neurosci. 30, 3640–3651. 10.1523/JNEUROSCI.4085-09.2010 20219998 PMC2872555

[B30] Simon O. Mangin J. F. Cohen L. Le Bihan D. Dehaene S. (2002). Topographical layout of hand, eye, calculation, and language-related areas in the human parietal lobe. Neuron 33, 475–487. 10.1016/s0896-6273(02)00575-5 11832233

[B31] Sivak M. (1996). The information that drivers use: is it indeed 90% visual? Perception 25, 1081–1089. 10.1068/p251081 8983048

[B306] Uchiyama Y. Toyoda H. Sakai H. Shin D. Ebe K. Sadato N. (2012). Suppression of brain activity related to a car-following task with an auditory task: an fMRI study. Trans. Res. Part Traff. Psychol. Behav. 15, 25–37. 10.1016/j.trf.2011.11.002

[B32] Wallach H. (1935). Über visuell wahrgenommene Bewegungsrichtung, Psychologische Forschung 20, 325–380. [translation into English with commentary by Wuerger, S., Shapley, R. and Rubin, N. (1996)]. On the visually perceived direction of motion. Perception 25, 1317–1367.

[B307] Wood J. M. (2002). Aging, driving and vision. Clin. Exp. Optom. 85, 214–220. 10.1111/j.1444-0938.2002.tb03040.x 12135413

[B33] Zeki S. Watson J. D. G. Lueck C. J. Friston K. J. Kennard C. Frackowiak R. S. J. (1991). A direct demonstration of functional specialization in human visual cortex. J. Neurosci. 11, 641–649. 10.1523/JNEUROSCI.11-03-00641.1991 2002358 PMC6575357

