# Supplementary material for: The Shape of a Vehicle Windshield Affects Reaction Time and Brain Activity During a Target Detection Task
Source: Front Hum Neurosci. 2020 May 26;14:183. doi: 10.3389/fnhum.2020.00183 (PMC7264157; doi:10.3389/fnhum.2020.00183)
Supplement: Supplementary file 1 [file Data_Sheet_1.docx]

Supplementary Material

Shape of a Vehicle Windshield affects Reaction Time and Brain Activity during a Target Detection Task

Takafumi Sasaoka*, Maro G. Machizawa, Yoshihisa Okamoto, Koji Iwase, Toshihiro Yoshida, Nanae Michida, Atsuhide Kishi, Masaki Chiba, Kazuo Nishikawa, Shigeto Yamawaki, Takahide Nouzawa

*** Correspondence:** Takafumi Sasaoka: tsasaoka@hiroshima-u.ac.jp

# Supplementary Tables

**Supplementary Table 1.** Results of four-way non-parametric ANOVA with the sum of optic flow as the dependent variable and *θ* (5°, 10°, 15°), *A* (10, 20, 30, 40, 50, 60 pixels), speed (fast, slow), and pillar (tilted, vertical) as independent factors.

|  | *df* | Sum of squares | Mean square | *F* | *R*^2^ | *p* |
| --- | --- | --- | --- | --- | --- | --- |
| *θ* | 2 | 54.54 | 27.27 | 5878 | 0.02767 | 0.001 |
| *A* | 5 | 551.08 | 110.22 | 23758 | 0.27963 | 0.001 |
| Speed | 1 | 782.67 | 782.67 | 168708 | 0.39714 | 0.001 |
| Pillar | 1 | 23.21 | 23.21 | 5002 | 0.01177 | 0.001 |
| *θ***A* | 10 | 23.53 | 2.35 | 507 | 0.01194 | 0.001 |
| *θ**Speed | 2 | 30.5 | 15.25 | 3287 | 0.01548 | 0.001 |
| *A**Speed | 5 | 316 | 63.2 | 13623 | 0.16034 | 0.001 |
| *θ**Pillar | 2 | 1.47 | 0.74 | 159 | 0.00075 | 0.001 |
| *A**Pillar | 5 | 30.09 | 6.02 | 1297 | 0.01527 | 0.001 |
| Speed*Pillar | 1 | 33.6 | 33.6 | 7244 | 0.01705 | 0.001 |
| *θ***A**Speed | 10 | 33.15 | 3.31 | 215 | 0.01682 | 0.001 |
| *θ***A**Pillar | 10 | 1.46 | 0.15 | 31 | 0.00074 | 0.001 |
| *θ**Speed*Pillar | 2 | 5.53 | 2.77 | 597 | 0.00281 | 0.001 |
| *A**Speed*Pillar | 5 | 9.79 | 1.96 | 422 | 0.00497 | 0.001 |
| *θ***A**Speed*Pillar | 10 | 1.34 | 0.13 | 29 | 0.00068 | 0.001 |
| Residuals | 15696 | 72.82 | 0 |  | 0.03695 | 0.001 |
| Total | 15767 | 1970.79 |  |  | 1 |  |

**Supplementary Table 2.** Results of four-way non-parametric ANOVA with the mean difference in optic flow between the left (without a pillar) and right (with a pillar) sides of the windshield as the dependent variable and *θ* (5°, 10°, 15°), *A* (10, 20, 30, 40, 50, 60 pixels), speed (fast, slow), and pillar (tilted, vertical) as independent factors.

|  | *df* | Sum of squares | Mean square | *F* | *R*^2^ | *p* |
| --- | --- | --- | --- | --- | --- | --- |
| *θ* | 2 | 18.41 | 9.203 | 6737 | 0.03336 | 0.001 |
| *A* | 5 | 165.02 | 33.004 | 24160 | 0.29907 | 0.001 |
| Speed | 1 | 280.41 | 280.408 | 205267 | 0.50819 | 0.001 |
| Pillar | 1 | 1.86 | 1.861 | 1363 | 0.00337 | 0.001 |
| *θ***A* | 10 | 2.23 | 0.223 | 163 | 0.00405 | 0.001 |
| *θ**Speed | 2 | 5.15 | 2.576 | 1886 | 0.00934 | 0.001 |
| *A**Speed | 5 | 45.53 | 9.107 | 6666 | 0.08252 | 0.001 |
| *θ**Pillar | 2 | 0.55 | 0.273 | 200 | 0.00099 | 0.001 |
| *A**Pillar | 5 | 5.24 | 1.047 | 767 | 0.00949 | 0.001 |
| Speed*Pillar | 1 | 2.31 | 2.307 | 1689 | 0.00418 | 0.001 |
| *θ***A**Speed | 10 | 1.67 | 0.167 | 122 | 0.00302 | 0.001 |
| *θ***A**Pillar | 10 | 0.32 | 0.032 | 23 | 0.00058 | 0.001 |
| *θ**Speed*Pillar | 2 | 0.78 | 0.392 | 287 | 0.00142 | 0.001 |
| *A**Speed*Pillar | 5 | 0.71 | 0.143 | 105 | 0.00129 | 0.001 |
| *θ***A**Speed*Pillar | 10 | 0.15 | 0.015 | 11 | 0.00027 | 0.001 |
| Residuals | 15696 | 21.44 | 0.001 |  | 0.03886 |  |
| Total | 15767 | 551.78 |  |  | 1 |  |

**Supplementary Table 3.** Results of three-way repeated-measures ANOVA (*N* = 34) with factors of vehicle speed, pillar, and target position for mean RTs in each condition. **(A)** The table of the three-way repeated-measures ANOVA. **(B)** The results of tests for simple main effects of the factors of speed and pillar and simple interaction of these factors.

**(A)**

|  | Sum of squares | Mean square | Mean square error | corrected *df*_1_ | corrected *df*_2_ | *F* | Partial *η*^2^ | *p* |  |
| --- | --- | --- | --- | --- | --- | --- | --- | --- | --- |
| Position | 0.3093 | 0.07734 | 0.00080 | 2.73 | 90.04 | 97.24 | .747 | .000 | ** |
| Speed | 0.0210 | 0.02099 | 0.00192 | 1 | 33 | 10.93 | .249 | .002 | ** |
| Pillar | 0.0017 | 0.00172 | 0.00154 | 1 | 33 | 1.11 | .033 | .299 |  |
| Positon*Speed | 0.0018 | 0.00045 | 0.00026 | 3.12 | 102.80 | 1.77 | .051 | .155 |  |
| Position*Pillar | 0.0042 | 0.00106 | 0.00032 | 3.09 | 101.89 | 3.25 | .090 | .024 | * |
| Speed*Pillar | 0.0013 | 0.00135 | 0.00193 | 1 | 33 | 0.70 | .021 | .410 |  |
| Positon*Speed*  Pillar | 0.0023 | 0.00058 | 0.00033 | 3.28 | 108.23 | 1.74 | .050 | .159 |  |

**(B)**

|  |  | Sum of squares | Mean square | Mean square error | *df*_1_ | *df*_2_ | *F* | Partial *η*^2^ | *p* |  |
| --- | --- | --- | --- | --- | --- | --- | --- | --- | --- | --- |
| Far-left | Speed | 0.00771 | 0.00771 | 0.00059 | 1 | 165 | 13.09 | .284 | .000 | ** |
|  | Pillar | 0.00224 | 0.00224 | 0.00057 | 1 | 165 | 3.94 | .107 | .049 | * |
|  | Speed*Pillar | 0.00035 | 0.00035 | 0.00065 | 1 | 165 | 0.54 | .005 | .464 |  |
| Near-left | Speed | 0.00581 | 0.00581 | 0.00059 | 1 | 165 | 9.87 | .230 | .002 | ** |
|  | Pillar | 0.00000 | 0.00000 | 0.00057 | 1 | 165 | 0.00 | .000 | .964 |  |
|  | Speed*Pillar | 0.00092 | 0.00092 | 0.00065 | 1 | 165 | 1.40 | .014 | .238 |  |
| Center | Speed | 0.00105 | 0.00105 | 0.00059 | 1 | 165 | 1.78 | .051 | .184 |  |
|  | Pillar | 0.00072 | 0.00072 | 0.00057 | 1 | 165 | 1.26 | .037 | .263 |  |
|  | Speed*Pillar | 0.00164 | 0.00164 | 0.00065 | 1 | 165 | 2.51 | .024 | .115 |  |
| Near-right | Speed | 0.00503 | 0.00503 | 0.00059 | 1 | 165 | 8.54 | .206 | .004 | ** |
|  | Pillar | 0.00059 | 0.00059 | 0.00057 | 1 | 165 | 1.04 | .031 | .309 |  |
|  | Speed*Pillar | 0.00023 | 0.00023 | 0.00065 | 1 | 165 | 0.36 | .004 | .550 |  |
| Far-right | Speed | 0.00321 | 0.00321 | 0.00059 | 1 | 165 | 5.45 | .142 | .021 | * |
|  | Pillar | 0.00239 | 0.00239 | 0.00057 | 1 | 165 | 4.20 | .113 | .042 | * |
|  | Speed*Pillar | 0.00052 | 0.00052 | 0.00065 | 1 | 165 | 0.80 | .008 | .374 |  |

**Supplementary Table 4.** Results of three-way repeated-measures ANOVA (*N* = 31) with factors of vehicle speed, pillar, and target position for mean RTs in each condition. **(A)** The table of the three-way repeated-measures ANOVA. **(B)** The results of tests for simple main effects of the factors of speed and pillar and simple interaction of these factors.

**(A)**

|  | Sum of squares | Mean square | Mean square error | corrected *df*_1_ | corrected *df*_2_ | *F* | Partial *η*^2^ | *p* |  |
| --- | --- | --- | --- | --- | --- | --- | --- | --- | --- |
| Position | 0.27616 | 0.06904 | 0.00078 | 2.61 | 78.39 | 88.61 | .747 | .000 | ** |
| Speed | 0.01913 | 0.01913 | 0.00194 | 1 | 30 | 9.88 | .248 | .004 | ** |
| Pillar | 0.00118 | 0.00118 | 0.00159 | 1 | 30 | 0.74 | .024 | .396 |  |
| Positon*Speed | 0.00179 | 0.00045 | 0.00027 | 3.04 | 91.13 | 1.67 | .053 | .178 |  |
| Position*Pillar | 0.00340 | 0.00085 | 0.00033 | 2.99 | 89.57 | 2.59 | .079 | .058 | + |
| Speed*Pillar | 0.00141 | 0.00141 | 0.00211 | 1 | 30 | 0.67 | .022 | .421 |  |
| Positon*Speed*  Pillar | 0.00196 | 0.00049 | 0.00032 | 3.17 | 95.10 | 1.53 | .048 | .211 |  |

**(B)**

|  |  | Sum of squares | Mean square | Mean square error | *df*_1_ | *df*_2_ | *F* | Partial *η*^2^ | *p* |  |
| --- | --- | --- | --- | --- | --- | --- | --- | --- | --- | --- |
| Far-left | Speed | 0.00800 | 0.00800 | 0.00060 | 1 | 150 | 13.30 | 0.307 | 0.0004 | ** |
|  | Pillar | 0.00184 | 0.00184 | 0.00058 | 1 | 150 | 3.17 | 0.096 | 0.0769 | + |
|  | Speed*Pillar | 0.00033 | 0.00033 | 0.00068 | 1 | 150 | 0.48 | 0.005 | 0.489 |  |
| Near-left | Speed | 0.00492 | 0.00492 | 0.00060 | 1 | 150 | 8.18 | 0.214 | 0.0049 | ** |
|  | Pillar | 0.00000 | 0.00000 | 0.00058 | 1 | 150 | 0.00 | 0.000 | 0.9576 |  |
|  | Speed*Pillar | 0.00100 | 0.00100 | 0.00068 | 1 | 150 | 1.48 | 0.016 | 0.2262 |  |
| Center | Speed | 0.00104 | 0.00104 | 0.00060 | 1 | 150 | 1.72 | 0.054 | 0.1916 |  |
|  | Pillar | 0.00063 | 0.00063 | 0.00058 | 1 | 150 | 1.08 | 0.035 | 0.2994 |  |
|  | Speed*Pillar | 0.00126 | 0.00126 | 0.00068 | 1 | 150 | 1.86 | 0.020 | 0.1751 |  |
| Near-right | Speed | 0.00413 | 0.00413 | 0.00060 | 1 | 150 | 6.86 | 0.186 | 0.0097 | ** |
|  | Pillar | 0.00033 | 0.00033 | 0.00058 | 1 | 150 | 0.56 | 0.018 | 0.4544 |  |
|  | Speed*Pillar | 0.00036 | 0.00036 | 0.00068 | 1 | 150 | 0.53 | 0.006 | 0.4674 |  |
| Far-right | Speed | 0.00284 | 0.00284 | 0.00060 | 1 | 150 | 4.72 | 0.136 | 0.0315 | * |
|  | Pillar | 0.00178 | 0.00178 | 0.00058 | 1 | 150 | 3.06 | 0.093 | 0.0822 | + |
|  | Speed*Pillar | 0.00042 | 0.00042 | 0.00068 | 1 | 150 | 0.61 | 0.007 | 0.4351 |  |

**Supplementary Table 5.** Results of functional magnetic resonance imaging of anatomical regions, peak voxel coordinates, and *t*-values for brain regions activated more for the tilted pillar than for the vertical pillar when a target was presented at the far-right position.

| **Anatomical region** | **Brodmann area** | **Cluster size** | **MNI coordinates (mm)** | | | ***t*-value** |
| --- | --- | --- | --- | --- | --- | --- |
|  |  |  | **x** | **y** | **z** |  |
| Left precuneus | 31 | 132 | -18 | -55 | 22 | 5.75 |
| Left posterior cingulate | 30 |  | -9 | -55 | 18 | 4.89 |
| Left precuneus | 31 |  | -9 | -67 | 30 | 4.68 |

Positive effect of conditions: uncorrected *p* < .001 at peak level, family-wise error corrected *p* < .05 at cluster level.

**Supplementary Table 6.** **(A)** Results of functional magnetic resonance imaging of anatomical regions, peak voxel coordinates, and *t*-values for brain regions activated more for the target presented at the far-right position than at the far-left position in the tilted pillar condition. **(B)** Results of functional magnetic resonance imaging of anatomical regions, peak voxel coordinates, and *t*-values for brain regions activated more for the target presented at the far-left position than at the far-right position in the tilted pillar condition.

**(A)**

| **Anatomical region** | **Brodmann area** | **Cluster size** | **MNI coordinates (mm)** | | | ***t*-value** |
| --- | --- | --- | --- | --- | --- | --- |
|  |  |  | **x** | **y** | **z** |  |
| Left middle temporal gyrus | 19/39 | 1472 | -42 | -73 | 14 | 8.39 |
| Left middle occipital gyrus | 19 |  | -45 | -73 | 2 | 7.25 |
| Left middle frontal gyrus | 9 | 206 | -42 | 11 | 30 | 5.19 |
| Left precentral gyrus | 6 |  | -36 | 5 | 30 | 5.18 |
| Left middle frontal gyrus | 6 |  | -39 | -1 | 46 | 4.97 |

**(B)**

| **Anatomical region** | **Brodmann area** | **Cluster size** | **MNI coordinates (mm)** | | | ***t*-value** |
| --- | --- | --- | --- | --- | --- | --- |
|  |  |  | **x** | **y** | **z** |  |
| Right fusiform gyrus | 37 | 276 | 27 | -64 | -10 | 7.11 |
| Right middle temporal gyrus | 37 |  | 45 | -64 | 2 | 6.40 |
| Right fusiform gyrus | 37 |  | 42 | -52 | -18 | 4.32 |

Uncorrected *p* < .001 at peak level, family-wise error corrected *p* < .05 at cluster level.

**Supplementary Table 7.** Results of functional magnetic resonance imaging of anatomical regions, peak voxel coordinates, and *F*-values for activations related to amount of upward optic flow normalized across all four conditions when the target was presented at the far-right position in all speed and pillar conditions

| **Anatomical region** | **Brodmann area** | **Cluster size** | **MNI coordinates (mm)** | | | ***t*-value** |
| --- | --- | --- | --- | --- | --- | --- |
|  |  |  | **x** | **y** | **z** |  |
| Left middle temporal cortex | 19 | 2627 | -48 | -64 | 10 | 10.48 |
| Left precentral gyrus | 6 |  | -39 | -4 | 50 | 8.67 |
| Left precentral gyrus | 6 |  | -48 | -1 | 46 | 7.39 |
| Left lingual gyrus | 19 | 1049 | -24 | -61 | -14 | 7.77 |
| Right cerebellum |  |  | 18 | -52 | -22 | 6.36 |
| Left cerebellum |  |  | -33 | -55 | -26 | 6.30 |
| Right superior temporal gyrus | 22 | 390 | 63 | -40 | 18 | 6.26 |
| Right angular gyrus | 39 |  | 42 | -49 | 42 | 5.24 |
| Right supramarginal gyrus | 40 |  | 45 | -40 | 42 | 5.06 |
| Left central operculum | 6 | 198 | -45 | -4 | 14 | 5.95 |
| Left frontal operculum | 44 |  | -48 | 14 | 2 | 4.71 |
| Left opercular part of inferior frontal gyrus | 44 |  | -60 | 14 | 18 | 4.41 |

Positive effect of conditions: uncorrected *p* < .001 at peak level, family-wise error corrected *p* < .05 at cluster level.

**Supplementary Table 8.** Results of functional magnetic resonance imaging of anatomical regions, peak voxel coordinates, and *F*-values for the far-right target onset-specific OF-related activity exclusively masked by the brain activity for target detection activations

| **Anatomical region** | **Brodmann area** | **Cluster size** | **MNI coordinates (mm)** | | | ***t*-value** |
| --- | --- | --- | --- | --- | --- | --- |
|  |  |  | **x** | **y** | **z** |  |
| Left middle temporal cortex | 19 | 2129 | -48 | -64 | 10 | 11.55 |
| Left precentral gyrus | 6 |  | -39 | -4 | 50 | 9.64 |
| Left superior temporal gyrus | 22 |  | -51 | -43 | 14 | 7.52 |
| Left precuneus | 7 |  | -9 | -55 | -58 | 5.56 |
| Left lingual gyrus | 19 | 601 | -24 | -61 | -14 | 8.89 |
| Right cerebellum |  |  | -12 | -70 | -22 | 6.32 |
| Left cerebellum |  |  | -42 | -64 | -30 | 5.47 |
| Left precentral gyrus | 6 | 473 | -48 | -1 | 6 | 5.82 |
| Left precentral gyrus | 6 |  | -42 | -1 | 14 | 5.63 |
| Left thalamus |  |  | -15 | -16 | 14 | 4.87 |

Uncorrected *p* < .001 at peak level, family-wise error corrected *p* < .05 at cluster level.

# Supplementary Figures

**
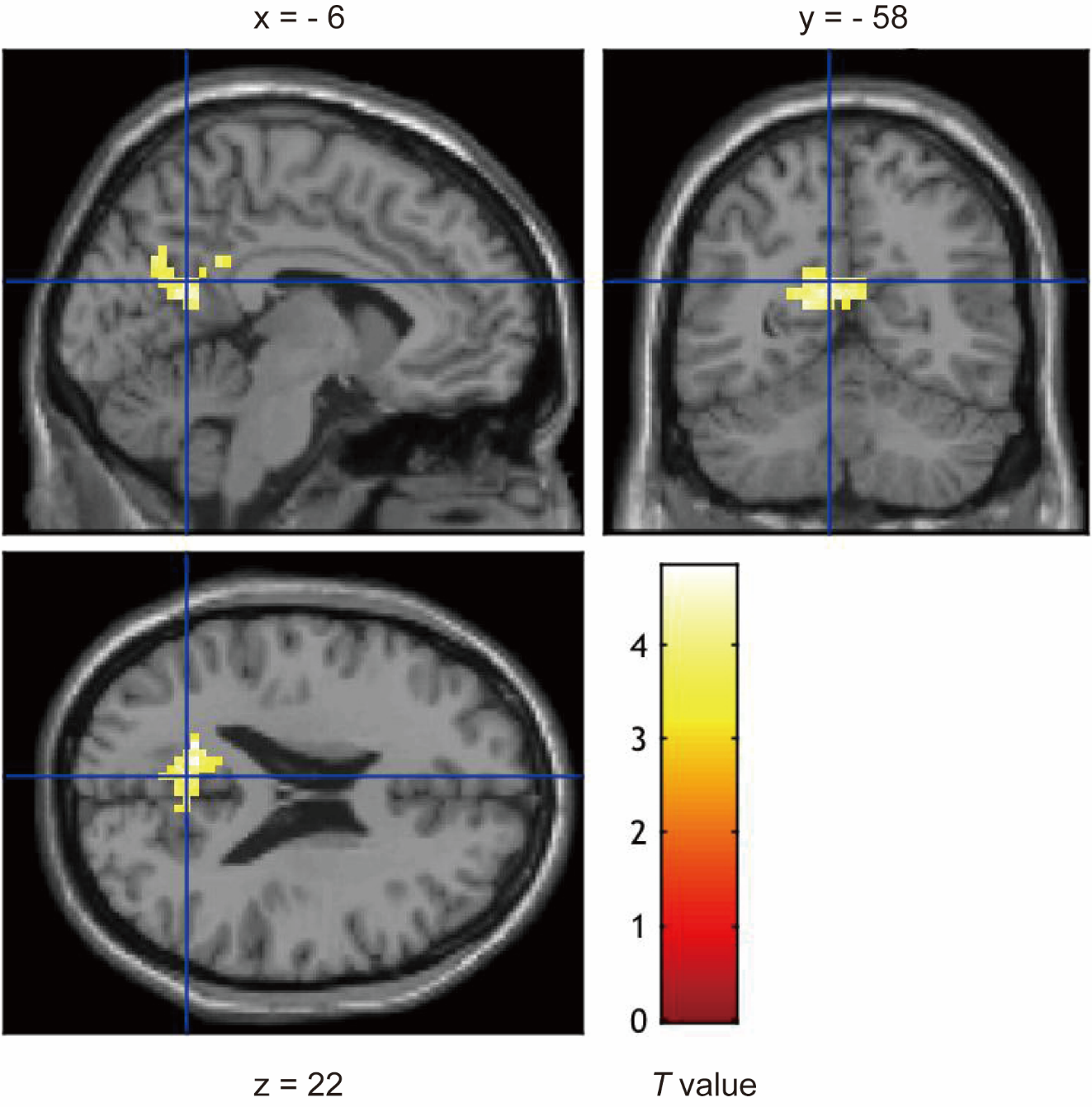
**

**Supplementary Figure 1.** Brain regions activated more for the tilted pillar than for the vertical pillar when a far-right target was presented.


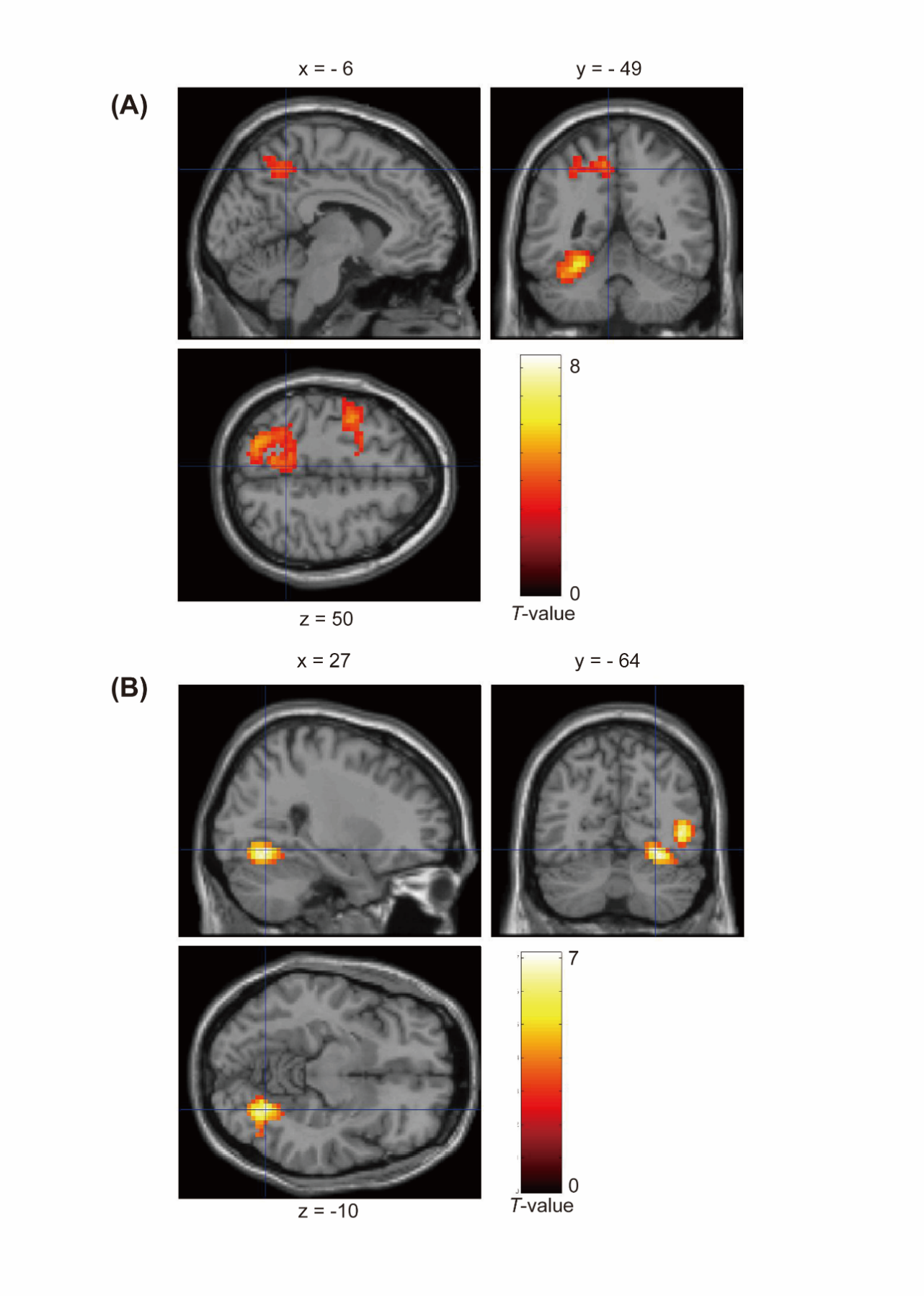


**Supplementary Figure 2.** The result of comparison of the brain activity between the far-left and far-right positions for the tilted pillar. **(A)** Brain regions activated more for the far-right target than for the far-left target in the tilted pillar condition. **(B)** Brain regions activated more for the far-left target than for the far-right target in the tilted pillar condition.


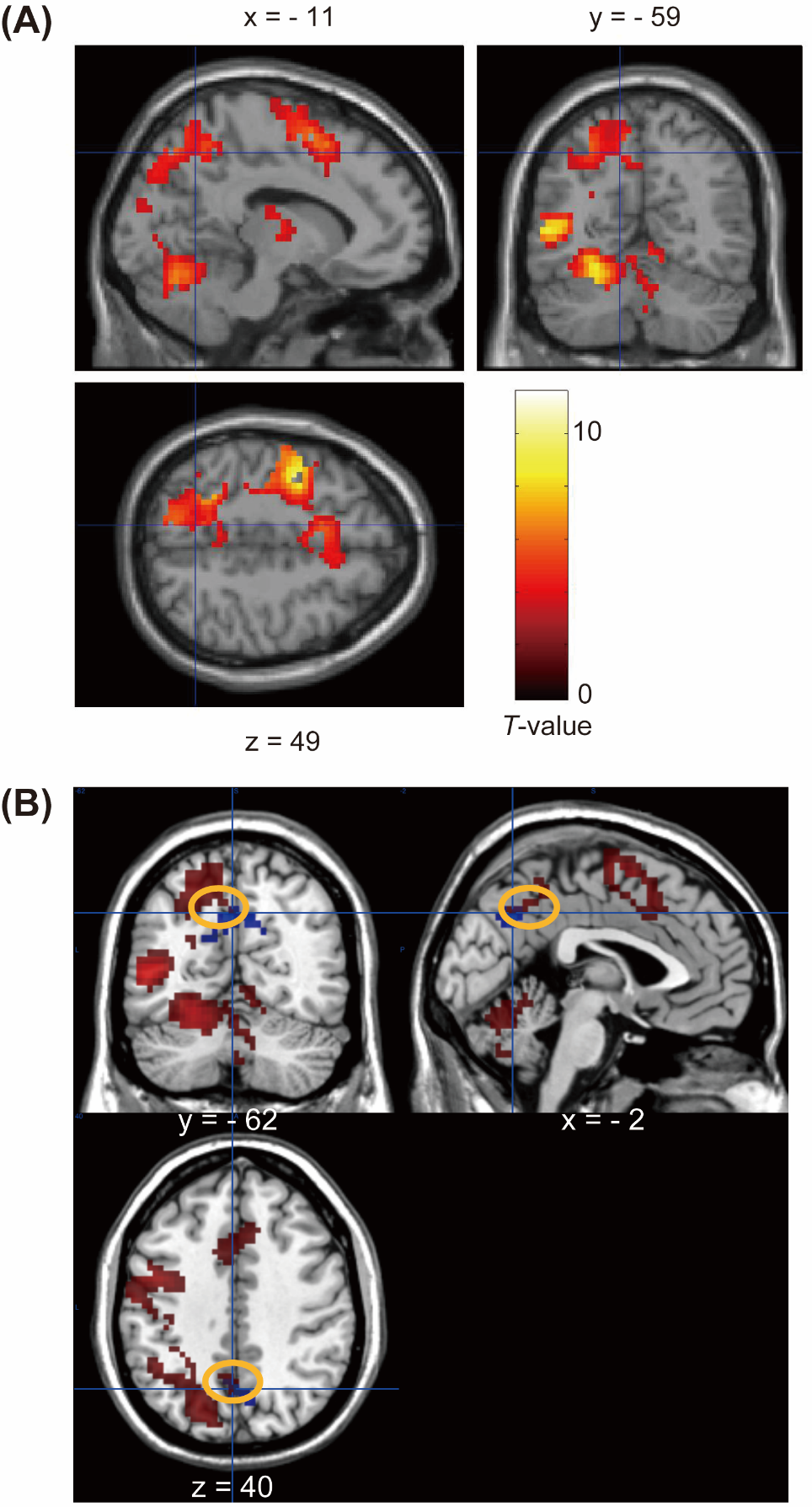


**Supplementary Figure 3.** **(A)** Brain regions showing the far-right target onset-specific OF-related activity exclusively masked by the brain activity for target detection. **(B)** Overlapping activation within the precuneus (the area indicated by an orange line) between the tilted pillar-related and the far-right target onset-specific OF-related activity exclusively masked by the brain activity for target detection (uncorrected *p* < .001 at peak level, family-wise error corrected *p* < .05 at cluster level). Red: activated regions showing the interaction between pillar and target position. Blue: regions showing the far-right target onset-specific OF-related activity exclusively masked by the brain activity for target detection.
